# Substygophily in Dinaric Karst: A Model Case of Locally Endemic Minnows *Phoxinellus* (Leuciscinae)

**DOI:** 10.1002/ece3.70648

**Published:** 2024-12-23

**Authors:** Anja Palandačić, Susanne Reier, Oleg A. Diripasko, Dušan Jelić, Andrej Stroj, Alexandra Wanka, Dario Marić, Nina G. Bogutskaya

**Affiliations:** ^1^ First Zoological Department Vienna Museum of Natural History Vienna Austria; ^2^ Department of Biology, Biotechnical Faculty University of Ljubljana Ljubljana Slovenia; ^3^ Institute of Fisheries and Marine Ecology Berdyansk Ukraine; ^4^ Croatian Institute for Biodiversity Zagreb Croatia; ^5^ Croatian Geological Survey Zagreb Croatia; ^6^ Dobrič b.b. Široki Brijeg Bosnia and Herzegovina; ^7^ BIOTA j d.o.o. Dolga Gora Slovenia

**Keywords:** ecology, fish, historical collections, molecular analysis, morphology, paleohydrology, subterranean

## Abstract

The Dinaric Karst extends along the Adriatic coast of the Western Balkan Peninsula and is home to a group of “karst minnows” of the genera *Delminichthys*, *Phoxinellus*, and *Telestes*, which have adapted to the highly variable water conditions in the karst by spending up to several months underground, but require surface habitats for spawning, defining them as substygophiles. The three species of the genus *Phoxinellus*, *P. alepidotus* , *P. pseudalepidotus*, and *P. dalmaticus* , are defined by restricted ranges, making them vulnerable to pollution and extended draughts caused by the climate change. In this study, the phylogeny of Leusciscinae was reconstructed using 15 *Phoxinellus* and one *Delminichthys adspersus*, one *Pelasgus epiroticus*, and one *Telestes polylepis* complete mitochondrial genomes and the position of the genus *Phoxinellus* within the subfamily as sister species to the *Chondrostoma* clade was confirmed. The inter‐ and intrapopulation structure of the genus *Phoxinellus* was inferred using molecular (nuclear and mitochondrial data) and morphological analyses. For the molecular analysis, more than 150 historical specimens were analyzed for a short fragment of the cytochrome oxidase I (COI) barcoding region and 15 *Phoxinellus* specimens were subjected to single nucleotide polymorphism analysis. For morphological analysis, 121 *Phoxinellus* specimens were analyzed for 51 measurements and 8 counts. All analyses confirmed the clear delimitation of the three *Phoxinellus* species, but were insufficient to fully resolve the intrapopulation structure within the species. This study also included data from field surveys of *Phoxinellus* collected over the past 20 years, which showed that abundance is declining and ranges are shrinking. *Phoxinellus* are also threatened by invasive/introduced species. Based on cave observations/occurrence and morphological analysis, *P. dalmaticus* was classified as an advanced substygophile and *P. alepidotus* and *P. pseudalepidotus* were classified as basic stygophiles.

## Graphical abstract

*Phoxinellus* minnows have adapted to the highly variable water conditions of karst poljes, ranging from floods to draughts within shorter (seasonal, annual) and longer (decadal) periods. In this study, based on the analysis of their morphological characters and their presence (or absence) in caves, they were classified more precisely, with *P. dalmaticus* as an advanced substygophile and *P. alepidotus* and *P. pseudalepidotus* as basic substygophiles. The reduction of their population size and distribution was documented herein. An integral approach of molecular and morphological analyses coupled with field observations and paleohydrology helped to elucidate different aspects of the biology of this locally endemic species, which will be crucial for their conservation. Finally, with the use of historical collections, we were able to include extinct populations of *P. alepidotus* in the analysis.

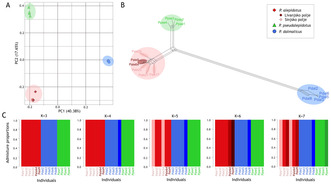

## 1 Introduction

The Dinaric Karst, part of the Dinaric Mountains, is the largest continuous karst area in Europe, stretching across several countries from Italy to northern Albania, parallel to the eastern Adriatic coast. It features typical karst phenomena, such as dolines and karst poljes above ground, and sinkholes, caves, and underground rivers below the ground. These features are formed by the ongoing process of karstification—water dissolving the soluble rock, resulting, among other things, in a complex water network connecting surface and underground water bodies. Karst poljes are closed plains that mostly drain underground, featuring perennial or intermittent sinking streams (Gams 1978). They are usually prone to flooding and can temporarily become lakes (partially or completely). Thus, the water conditions of poljes are highly variable, ranging from floods to draughts within shorter (seasonal, annual) and longer (decadal) periods (for details see Bonacci 2013).

A group of “karst minnows” of the genera *Delminichthys*, *Phoxinellus*, and *Telestes* have adapted to the highly variable water conditions of karst poljes, and while they can spend up to several months underground, they require surface habitats for spawning (Trgovčević 1905; Ćurčić 1913; Vuković and Ivanović 1971; Vuković 1977). Therefore, they were characterized as stygophiles by Jelić, Špelić, and Žutinić (2016). However, stygophily applies to aquatic species with subterranean and epigean populations, whereas the term subtroglophile is defined based on the requiring surface environment for at least one vital function (Sket 2008) and is therefore more applicable to these genera. Yet, as “troglo” applies to terrestrial organisms, and “stygo” to aquatic organisms, the term substygophile was adopted in this study (see also Section 4). The genera *Delminichthys*, *Phoxinellus*, and *Telestes* lack obvious cave adaptations such as reduced eyes and pigmentation, though they exhibit other traits that may be related to their partially subterranean lifestyle, such as some thickening of the skin, reduced cephalic sensory canals, and reduction of scales (Bogutskaya and Zupančič 2003; Freyhof et al. 2006). The species of the three genera are cold‐adapted species with similar dietary preferences (Mrakovčić et al. 2006; Zanella et al. 2009; Markotić et al. 2019; Marčić et al. 2021), but detailed data are still needed. All of them have restricted ranges, with species mostly occupying small springs of one to several karst poljes. Originally placed in the genus *Phoxinellus*, a molecular study by Freyhof et al. (2006) revealed the paraphyletic status of the group and retained three species in the genus *Phoxinellus*, while others were placed either in *Telestes* or in a newly introduced genus *Delminichthys*. The phylogenetic relationships of the species within the genera and their position in Leuciscinae have since been confirmed in several molecular studies (Palandačić, Zupančič, and Snoj 2010; Perea et al. 2010; Schönhuth et al. 2018), but they were mostly based on single genes and often lacked sufficient statistical support.

**FIGURE 1:**
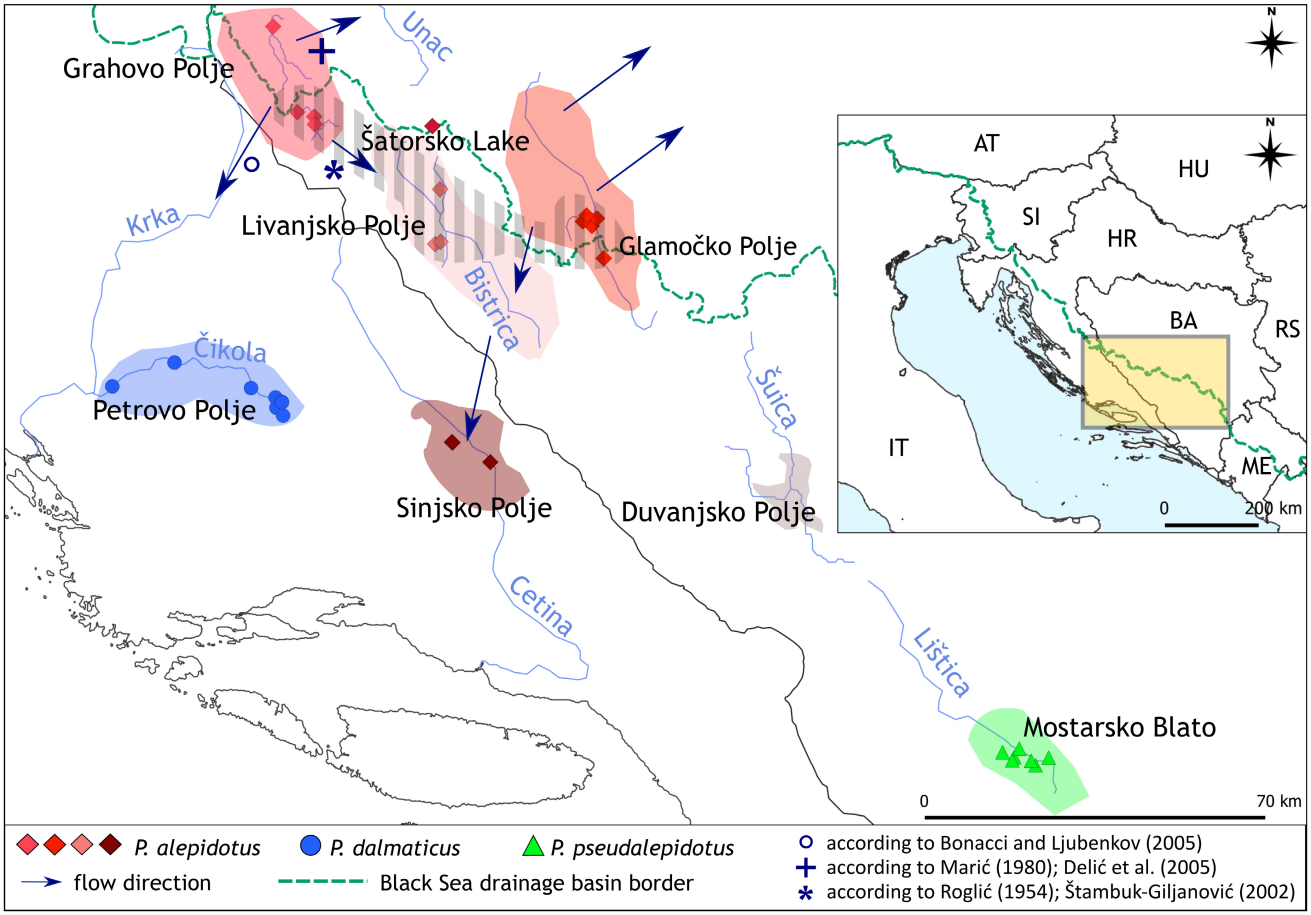
A schematic representation of the study area with its poljes. The study area is located in the central part of the Dinaric Karst on the territory of Croatia and Bosnia‐Herzegovina. Three different *Phoxinellus* species are represented with different symbols and colors. The simplified superficial and underground water flows between four poljes inhabited by *Phoxinellus alepidotus* is shown with arrows. According to Roglić (1954) and Štambuk‐Giljanović (2002), waters from Grahovo Polje drain into Cetina River (marked with a star); according to Bonacci and Ljubenkov (2005) into the upper Krka River (marked with a circle); and according to Marić (1980) and Delić et al. (2005) to Una River (Danube) (marked with a cross). In the species range summarized from all available old and recent literature, five poljes are given: Grahovo, Livanjsko, Glamočko, Sinjsko, and Duvanjsko. However, there were never any confirmed fish specimen, historical or recent, collected at Duvanjsko Polje. Despite field surveys, *Phoxinellus* in Sinjsko Polje were not observed in the last 20 years, thus are believed to be extirpated from this polje by the authors of this study. The species range of *P. alepidotus* observed in this study is marked with gray stripes.

Based on (single gene) molecular (Freyhof et al. 2006; Palandačić, Zupančič, and Snoj 2010; Perea et al. 2010; Schönhuth et al. 2018) and morphological analyses (Zupančič and Bogutskaya 2000; Bogutskaya and Zupančič 2003), the three species of the genus *Phoxinellus*— *P. alepidotus* (Heckel 1843); *P. pseudalepidotus* Bogutskaya and Zupančič 2003; and *P. dalmaticus* Zupančič and Bogutskaya 2000 are well delimited from each other and form their sister group, the *Chondrostoma* clade (Schönhuth et al. 2018). They are defined by restricted species ranges (Figure 1, Table 1), with *P. pseudalepidotus* occurring only in the system of a single, 10 km long river in the karst polje Mostarsko Blato (Bosnia‐Herzegovina) and *P. dalmaticus* in the upper reaches of the Čikola River (Petrovo Polje, Croatia), with an area of occupancy of about 20 km^2^ (Bonacci, Terzić, and Roje‐Bonacci 2018; Andabaka, Senta, and Gudelj 2021). *Phoxinellus alepidotus* is comparatively more widespread, having been reported in the literature from five adjacent karst poljes in Croatia and Bosnia‐Herzegovina. Their extremely limited distributions make them vulnerable to extinction, as even a single pollution event could wipe out the population and thus the entire species. Furthermore, in other karst poljes, the endemic fish fauna is threatened by introduced and invasive species such as *Salmo* sp., *Micropterus salmoides* and *Lepomis gibbosus* (Jelić, Špelić, and Žutinić 2016), which could potentially also pose a problem for *Phoxinellus*. Additional threats are the effects of the climate change and increased demand for water use in the Dinaric Karst (Bonacci and Roje‐Bonacci 2003; Bonacci 2004; Bonacci, Buzjak, and Roje‐Bonacci 2016; Bonacci et al. 2020).

**TABLE 1:** Species ranges of *Phoxinellus* species, based on all available literature, with basic descriptions of the poljes, they occupy. Comments on their current distribution are also given.

Species of *Phoxinellus*	Polje	Main river	Drains into	SDB	Anthropogenic influence	Remarks on current distribution
*dalmaticus*	Petrovo	Čikola	Krka R near Torak Lake	Adriatic	Water pumping station built at the end of the 1980s supplies water to the town Drniš	Latest confirmation in 2018 (personal observation DJ)
*alepidotus*	Grahovo	Korana	According to Roglić (1954), Štambuk‐Giljanović (2002) into Cetina R; according to Bonacci and Ljubenkov (2005) into the upper Krka R; according to Marić (1980) and Delić et al. (2005) to Una R (Danube R)	Adriatic/Black		Last published record Geiger et al. (2014), but samples probably originating from Freyhof et al. (2006). Confirmed observation by DJ in 2008
	Glamočko	Ribnik, Jaruga, Vrba	Southern part into Cetina R; central part into Pliva R (tributary to Vrbas R, Danube R); northern part into Sana R (tributary to Una R, Danube R)	Adriatic/Black		Last published record 2001 (Bogutskaya and Zupančič 2003); latest record 2009 (P. Zupančič, pers. comm.)
	Duvanjsko	Šujica	Underground to Livanjsko Polje	Adriatic		Appears as part of the range in literature (Ćurčić 1916; Taler 1953; Sabioncello 1967; Vuković and Ivanović 1971; Vuković 1977, 1982), but no historic or recent specimens known
	Livanjsko	Bistrica	Underground into Sinjsko Polje	Adriatic	Only 6.3 km of Bistrica free‐flowing, after that regulated, artificial riverbed; two artificial reservoirs for the hydropower plant	Last observed in 2016 by DJ
	Sinjsko	Cetina	Adriatic	Adriatic	Periodical flooding until Peruća hydropower plant was constructed in 1958	Historic sampling sites, last published in 2015, but possibly referring to the literature, not confirmed in the surveys since 1999
	Šatorsko Lake		Unac R, tributary of Una R (Sava R–Danube R)	Black	Introduced population	Introduced in 1970–1980 (Delić et al. 2005), last observation 2013 by DJ
*pseudalepidotus*	Mostarsko Blato	Lištica	Underground to Neretva R	Adriatic	Hydropower plant, for which two artificial canals and two artificial lakes were created	

Abbreviations: R, river; SDB, sea drainage basin.

According to Perea et al. (2010) and Reier, Bogutskaya, and Palandačić (2022), who performed divergence time estimates based on the phylogenetic reconstruction of the mitochondrial (mt) cytochrome b gene, the three *Phoxinellus* species diverged around 2.6 mya. This may be related to the extensive remodeling of the drainage network (Tari and Pamić 1998), along with the shifting positions of the Black Sea and Adriatic Sea drainage basins, which may have created the barriers to gene flow and confined each species to its range.

In this article, (i) complete mitochondrial genomes of the three *Phoxinellus* species were assembled to revise their phylogenetic relationships within the genus and their phylogenetic position within the subfamily Leuciscinae. After inferring the position of *Phoxinellus* within Leusciscinae using complete mitochondrial phylogeny, the study focused on intraspecific and interspecific analysis of the genus. (ii) First, historical specimens, including locally extinct populations and type material, were analyzed for a short fragment of the cytochrome oxidase I (COI) barcoding region in order to assess genetic diversity within the populations. While mt genes are not suitable for in‐depth population analysis, selected DNA samples from recent years, as well as historical DNA samples with good DNA quality, were sent for low‐coverage whole‐genome sequencing and analyzed to infer interspecific structure. Despite the low number of DNA samples, analysis of intraspecific structure was also attempted. Morphological analysis was carried out at these two levels, inter‐ and intraspecific.

(iii) This study also includes data from field surveys collected during the studies and monitoring of *Phoxinellus* populations over the last 20 years, which were compared with previous distribution ranges from the literature in order to update their distribution and conservation implications. (iv) In order to deduce the subterranean affinities of the three *Phoxinellus* species, the results of the morphological analysis of the characters known to be associated with the colonization of the caves were combined with field observations of their hypogean occurrence. (v) Finally, all the data collected were placed in the context of known (paleo)hydrogeological events in the area, in order to infer the reasons for the currently observed distribution of the populations/species.

## 2 Materials and Methods

### 2.1 Description of Species Distribution Areas

The three species of the genus *Phoxinellus* are distributed in the central part of the Dinaric Karst in Croatia and Bosnia‐Herzegovina (Figure 1). Brief descriptions of all poljes mentioned in the literature as the part of the distribution area are given here.

The species range of *P. dalmaticus* is the Petrovo Polje with the Čikola River, which flows into the Krka River and thus into the Adriatic Sea drainage basin (see also Bonacci, Terzić, and Roje‐Bonacci 2018; Andabaka, Senta, and Gudelj 2021). Literature records for the distribution of *P. alepidotus* include five karst poljes, Grahovo, Glamočko, Livanjsko, Sinjsko, and Duvanjsko, but for the latter, only literature accounts are given (Ćurčić 1916; Taler 1953; Sabioncello 1967; Vuković and Ivanović 1971; Vuković 1977, 1982), but no historical or recent voucher specimens exist. According to Roglić (1954), Štambuk‐Giljanović (2002) and Bonacci and Ljubenkov (2005), Grahovo Polje drains into Adriatic Sea drainage basin (either into the Cetina or the Krka River), but some authors (Marić 1980; Delić et al. 2005) report that the upper parts of Grahovo Polje drain toward northeast and thus into the Black Sea drainage basin. Glamočko Polje is hydrologically divided into three parts and drains into both the Adriatic and Black Sea drainage basins, while Duvanjsko, Sinjsko, and Livanjsko Polje drain into the Adriatic Sea drainage basin. *Phoxinellus alepidotus* has also been introduced outside of its native range into Šatorsko Lake (Delić et al. 2005), which belongs to the Black Sea drainage basin. The species range of *P. pseudalepidotus* is Mostarsko Blato with the Lištica River. Summary information on the poljes, their hydrology, and known anthropogenic interventions is given in Table 1, see also Figure 1. Additional descriptions can be found in the Supporting Information and in Table S1.

### 2.2 Sampling Methods

The historical specimens used in this study are from museum collections, thus the method of collection is unknown. Traditionally, these fish were collected using fish traps (Ćurčić 1913, 1916).

Field surveys were carried out on several occasions in 2008, 2015, 2016, 2018, and 2023 at sites across the species range and a very good perspective on events and changes over the past 15 years was gained. During this time, the *Phoxinellus* species and their ranges were surveyed using conventional fish survey methods, that is, electrofishing. SAMUS 765 (0.9kW) and Hans Grassl (1kW) backpack electrofishers were used for fish sampling in small streams, springs, and ponds. Since 2012, cave diving has been introduced as a method to reach animals that retreat underground during dry periods. During the cave dive, the main parameters inside the cave were measured and used to create a cave map. Distance from the entrance and profiles were measured with a tape measure, and depth, direction, and pitch angle were measured with a Suunto D9 dive computer. Temperature and pressure were also measured automatically with the Suunto D9 at 1‐min intervals throughout the dive.

### 2.3 Specimens Used for DNA Analysis

DNA extracts from previous studies were used for *P. dalmaticus* and *P. pseudalepidotus* (Reier, Bogutskaya, and Palandačić 2022). The term “recent” is used for the specimens collected within the last 30 years with the intention of DNA analysis. In addition, two historical specimens from the Fish Collection of the Natural History Museum in Vienna (NHMW), the holotype of *P. pseudalepidotus* collected in 1896 and *P. dalmaticus* collected in 1897, were included in the analysis (Table 2). For *P. alepidotus* , 148 historical specimens collected in 1854, 1856, 1881, and 1883, including possible syntypes (see Results section), were sampled and DNA extracted. Only those specimens that were successfully amplified for a partial fragment of COI are included in Table 2. Eight COI sequences from GenBank were also included (Table S2b). While the complete mitochondrial genomes of the Leuciscinae genera *Delminichthys*, *Pelasgus*, and *Telestes* were not yet available, DNA samples from *D. adspersus* , *Pe. epiroticus*, and *T. polylepis* were included in the dataset. The complete mitochondrial genomes downloaded from GenBank are listed in Table S2a (and Figure 4).

**TABLE 2:** Specimens of *Phoxinellus dalmaticus* , *P. alepidotus*, and *P. pseudalepidotus* used for DNA analysis. The historical specimens were collected in 1854, 1856, 1881, and 1883. The “recent” specimens were collected within the last 30 years with the intention of DNA analysis. See Table S2 for details.

Lab ID	Species	Historical/Recent	Fragment	GenBank no.	Polje	NMW number
Pale10	*alepidotus*	Historical	C1		Sinjsko	51110
Pale18	*alepidotus*	Historical	C1		Livanjsko	51057:2
Pale23	*alepidotus*	Historical	C1		Livanjsko	51057:7
Pale25	*alepidotus*	Historical	C1		Livanjsko	51057:9
Pale39	*alepidotus*	Historical	C1		Livanjsko	51059:3
Pale64	*alepidotus*	Historical	C1		Sinjsko	51108:1
Pale66	*alepidotus*	Historical	C1		Sinjsko	51108:3
Pale85	*alepidotus*	Historical	C1		Sinjsko	51062:6
Pale68	*alepidotus*	Historical	C2		Sinjsko	51108:5
Pale69	*alepidotus*	Historical	C2		Sinjsko	51108:6
Pale89	*alepidotus*	Historical	C2		Sinjsko	12971:1
Pale92	*alepidotus*	Historical	C2		Livanjsko	51058:3
Pale19	*alepidotus*	Historical	C1+C2	PQ289570	Livanjsko	51057:3
Pale22	*alepidotus*	Historical	C1+C2	PQ289573	Livanjsko	51057:6
Pale26	*alepidotus*	Historical	C1+C2	PQ289575	Livanjsko	51057:10
Pale27	*alepidotus*	Historical	C1+C2	PQ289576	Livanjsko	51057:11
Pale100	*alepidotus*	Historical	C1+C2	PQ289581	Sinjsko	51054:1
Pale101	*alepidotus*	Historical	C1+C2	PQ289582	Sinjsko	51054:2
Pale102	*alepidotus*	Historical	C1+C2	PQ289583	Sinjsko	51054:3
Pale103	*alepidotus*	Historical	C1+C2	PQ289584	Sinjsko	51054:4
Pale104	*alepidotus*	Historical	C1+C2	PQ289585	Sinjsko	51054:5
Pale105	*alepidotus*	Historical	C1+C2	PQ289586	Sinjsko	51054:6
Pale106	*alepidotus*	Historical	C1+C2	PQ289587	Sinjsko	51054:7
Pale107	*alepidotus*	historical	C1+C2	PQ289588	Sinjsko	51054:8
Pale108	*alepidotus*	Historical	C1+C2	PQ289589	Sinjsko	51054:9
Pale55	*alepidotus*	Historical	C1+C2	PQ289590	Sinjsko	51052:1
Pale56	*alepidotus*	Historical	C1+C2	PQ289591	Sinjsko	51052:2
Pale57	*alepidotus*	Historical	C1+C2	PQ289592	Sinjsko	51052:3
Pale58	*alepidotus*	Historical	C1+C2	PQ289593	Sinjsko	51052:4
Pale59	*alepidotus*	Historical	C1+C2	PQ289594	Sinjsko	51051:1
Pale60	*alepidotus*	Historical	C1+C2	PQ289595	Sinjsko	51051:2
Pale61	*alepidotus*	Historical	C1+C2	PQ289596	Sinjsko	51051:3
Pale62	*alepidotus*	Historical	C1+C2	PQ289597	Sinjsko	51051:4
Pale63	*alepidotus*	Historical	C1+C2	PQ289598	Sinjsko	51051:5
Pale65	*alepidotus*	Historical	C1+C2	PQ289600	Sinjsko	51108:2
Pale80	*alepidotus*	Historical	C1+C2	PQ289601	Sinjsko	51062:1
Pale81	*alepidotus*	Historical	C1+C2	PQ289602	Sinjsko	51062:2
Pale82	*alepidotus*	Historical	C1+C2	PQ289603	Sinjsko	51062:3
Pale83	*alepidotus*	Historical	C1+C2	PQ289604	Sinjsko	51062:4
Pale84	*alepidotus*	Historical	C1+C2	PQ289605	Sinjsko	51062:5
Pale93	*alepidotus*	Historical	C1+C2	PQ289606	Sinjsko	51111:1
Pale96	*alepidotus*	Historical	C1+C2	PQ289607	Sinjsko	51111:4
Pale99	*alepidotus*	historical	C1+C2	PQ289608	Sinjsko	51107:3
Pale64	*alepidotus*	Historical	CmtG	COI: PQ289599 CmtG: PQ431945 nt‐NCBI: SAMN44055087	Sinjsko	51108:1
Pale17	*alepidotus*	Historical	CmtG	COI: PQ315660 CmtG: PQ431941 nt‐NCBI: SAMN44055090	Livanjsko	51057:1
Pale9	*alepidotus*	Historical	CmtG	COI: PQ315659 CmtG: PQ431944 nt‐NCBI: SAMN44055088	Sinjsko	51110
Pale20	*alepidotus*	Historical	CmtG	COI: PQ289571 CmtG: PQ431940 nt‐NCBI: SAMN44055091	Livanjsko	51057:4
Pale24	*alepidotus*	Historical	CmtG	COI: PQ289574 CmtG: PQ431942 nt‐NCBI: SAMN44055089	Livanjsko	51057:8
Pale21	*alepidotus*	Historical	CmtG	COI: PQ289572 CmtG: PQ431943 nt‐NCBI: SAMN44055086	Livanjsko	51057:5
Pdal2	*dalmaticus*	Recent	CmtG	COI: PQ289565 CmtG: PQ431953 nt‐NCBI: SAMN44055077	Petrovo	Tissue only
Pdal3	*dalmaticus*	Recent	CmtG	COI: PQ289566 CmtG: PQ431954 nt‐NCBI: SAMN44055078	Petrovo	Tissue only
Pdal5	*dalmaticus*	Recent	CmtG	COI: PQ289567 CmtG: PQ431951 nt‐NCBI: SAMN44055079	Petrovo	Tissue only
Pdal6	*dalmaticus*	Recent	CmtG	COI: PQ289568 CmtG: PQ431952 nt‐NCBI: SAMN44055080	Petrovo	Tissue only
Pdal8	*dalmaticus*	Historical	CmtG	COI: PQ289569 CmtG: PQ431950 nt‐NCBI: SAMN44055081	Petrovo	51053
Ppse1	*pseudalepidotus*	Recent	CmtG	COI: PQ289577 CmtG: PQ431947 nt‐NCBI: SAMN44055082	Mostarsko Blato	Tissue only
Ppse2	*pseudalepidotus*	Recent	CmtG	COI: PQ289578 CmtG: PQ431949 nt‐NCBI: SAMN44055083	Mostarsko Blato	Tissue only
Ppse3	*pseudalepidotus*	Recent	CmtG	COI: PQ289579 CmtG: PQ431948 nt‐NCBI: SAMN44055084	Mostarsko Blato	Tissue only
Ppse4	*pseudalepidotus* Holotype	Historical	CmtG	COI: PQ289580 CmtG: PQ431946 nt‐NCBI: SAMN44055085	Mostarsko Blato	51102
OUTGROUPS						
NEZ09‐1	*Delminichthys adspersus*	Recent	CmtG	PQ431957		Tissue only
Pepir1	*Pelasgus epiroticus* Syntype	Historical	CmtG	PQ431956		51122
Tpol1	*Telestes polylepis* Paralectotype	Historical	CmtG	PQ431955		49710

Abbreviations: C1, only the first fragment of the cytochrome oxidase I (COI) was successfully amplified and sequenced; C2, only the second fragment of the COI was successfully amplified and sequenced; C1+C2, both fragments of COI were successfully sequenced; CmtG, complete mitochondrial genome was assembled; CmtG, GenBank number for complete mitochondrial genome; nt‐NCBI, raw nuclear data NCBI accession number.

### 2.4 Analysis of Mitochondrial DNA

Laboratory procedures were carried out in accordance with all requirements for working with historical museum material (historical DNA as defined by Raxworthy and Smith 2021), including the use of UV‐irradiated equipment, a clean room, and negative extraction controls (e.g., Fulton and Shapiro 2019). DNA was extracted from air‐dried gill rake tissue using the QIAamp DNA Blood and Tissue Micro Kit (Qiagen) according to the manufacturer's protocol. After DNA extraction, the amount of double‐stranded DNA was determined by fluorometry (Qubit; Thermo Fisher Scientific) using the double‐stranded DNA high‐sensitivity assay kit.

To reconstruct the phylogeny of Leusciscinae on the basis of complete mitochondrial genomes and to infer the position of the *Phoxinellus* genus within it (goal I), five *P. dalmaticus* , four *P. pseudalepidotus*, six *P. alepidotus* , one *D. adspersus* , one *Pe. epiroticus*, and one *T. polylepis* specimen were sent for paired end whole genome resequencing on the Illumina NovaSeq platform (IGA Technology Services, Udine, Italy). The raw sequences were then trimmed and complete mt genomes were assembled from a subset of 10 million pair‐end reads using Geneious v 10.2.6 (http://www.geneious.com; for details see Palandačić et al. 2023), allowing for a sufficient coverage of ≥ 10×. The subset of 10 million reads was first blasted to the reference, for which the complete mitochondrial genome of *Pseudochondrostoma polylepis* (NC_031574) was used. In a second step, the blasted reads were aligned to the same sequence (NC_031574).

The newly assembled complete mt genomes were aligned together with the complete mt genomes of other Leuciscinae genera downloaded from the GenBank (Table S2). *Tinca tinca* (NC_008648; as suggested by Schönhuth et al. 2018) was used as an outgroup. The alignment was then used for phylogenetic inference using maximum likelihood (ML) and Bayesian inference (BI) approaches implemented in the PhyloSuite v.1.2.2 software (Zhang et al. 2020). First, the alignment was realigned using MAFFT 7 (Katoh and Standley 2013) and the most appropriate evolutionary model was determined using PartitionFinder2 (Lanfear et al. 2017). Tree search was performed with IQtree (Nguyen et al. 2015) and MrBayes v.3.2.6 (Ronquist et al. 2012), all implemented in PhyloSuite.

Analysis of the COI barcode region was undertaken to infer inter‐ and intrapopulation structure (goal II). As historical DNA is typically fragmented (Raxworthy and Smith 2021), two overlapping fragments of the COI were amplified by polymerase chain reaction (PCR) using specific primer pairs FishF1 (Ward et al. 2005) and COI_mus_R1 (Palandačić et al. 2017) for the first fragment of COI (designated C1) and C2_Pel_1f and C2_Pel_1r (Tsaparis et al. 2011) for the second fragment of COI (designated C2). The overlapping fragments were aligned in MEGA 6 (Tamura et al. 2013) and concatenated into a single sequence. Furthermore, the alignment was used to construct a TCS (Templeton, Crandall, and Sing's) parsimonious haplotype network implemented in PopART v1.7 software (Leigh and Bryant 2015).

### 2.5 Analysis of the Nuclear DNA

To infer the inter‐ and intrapopulation structure based on nuclear DNA (goal II), the same 15 *Phoxinellus* DNA samples as listed earlier for the assembly of complete mt genomes were analyzed (Table 2). The analysis was based on the calculation of genotype frequencies of single nucleotide polymorphisms (SNPs) within and between the populations.

In the absence of a reference genome for the study organism, a de novo draft assembly was constructed using the raw reads from a *P. pseudalepidotus* (Ppse1) DNA sample with the highest read coverage. The raw reads were trimmed of low‐quality bases and adapter sequences using trim‐galore v0.6.2 (Krueger et al. 2023), leaving only high‐quality reads, which were then assembled using SPAdes v3.5.13 (Bankevich et al. 2012) with default parameters. SPAdes is a de novo assembler designed to effectively handle short read sequencing data, and despite low coverage, produces contigs for the construction of the draft assembly. BBMap v38.90 (Bushnell 2014) was used to evaluate the draft assembly. This involved running the stats.sh script to obtain assembly statistics. The raw reads of Ppse1 were then remapped to the draft assembly using BWA v0.7.13 (Li 2013), and the assembly was further evaluated using Qualimap v2.2.1 (Okonechnikov, Conesa, and García‐Alcalde 2016) for coverage analysis. BUSCO v5.4.3 (Manni et al. 2021) was used to assess the completeness of the genome assembly by searching for conserved single‐copy orthologs using the Actinopterygii dataset.

Sequencing reads from the 15 specimens were aligned to the draft assembly using BWA‐MEM v0.7.13 (Li 2013). This alignment generated SAM files, which were then converted to BAM format and sorted using SAMtools v1.18 (Danecek et al. 2021). Picard‐tools v2.27.5 (http://broadinstitute.github.io/picard/) was used to process the BAM files generated from the mapping step. Duplicate reads were marked (“MarkDuplicates”), mate information was fixed (“FixedMateInformation”), and reads groups were replaced (“AddOrReplaceReadGroups”).

Due to differences in sequencing depth and to account for possible sequencing errors, ANGSD (analysis of next generation sequencing data; Korneliussen, Albrechtsen, and Nielsen 2014) was used for downstream population genomic analyses. ANGSD's robust handling of low‐coverage data and its ability to work with draft assemblies made it a suitable choice for this study. Instead of performing hard genotyping, genotype likelihoods were calculated using the Samtools genotype likelihood model (Li 2011), and a file containing the output genotype likelihoods in beagle format was generated. SNPs were included if they were covered by at least three reads in 80% of individuals, passed a *p* value cut‐off of ×10^−6^ (Kim et al. 2011) for variability, and had no more than two different alleles. Reads that did not map uniquely to the reference had a mapping quality of < 30 or bases with a quality score of < 20 were excluded from genotype likelihood calculations in subsequent analyses. The following *p* value cut‐offs for single nucleotide polymorphism (SNP) filters were applied in ANGSD: ‐sb_pval 0.05, ‐qscore_pval 0.05, ‐edge_pval 0.05, ‐mapq_pval 0.05.

Further analyses were performed to explore the population structure and genetic relationships among the specimens. NGSadmix (Skotte, Korneliussen, and Albrechtsen 2013) was used to infer population structure by estimating the proportions of individual ancestry, with the number of ancestral populations (*K*) set from 2 to 6. PCangsd (Meisner and Albrechtsen 2018) was used to perform principal component analysis (PCA), which provides insight into the genetic variation and clustering of individuals using the default parameters. The BCF output file was converted to VCF format using bcftools (Danecek et al. 2021), which was then used to construct an unrooted phylogenetic network with the neighbor‐net algorithm and uncorrected p distances using SplitsTree v4.19.2 (Huson, Kloepper, and Bryant 2008). This analysis helped to visualize the genetic distances and relationships between the specimens, revealing the genetic structure within the species.

### 2.6 Specimens Used for Morphological Analysis

The morphological study was based on historical and recent samples listed in Zupančič and Bogutskaya (2000) and Bogutskaya and Zupančič (2003). In addition, recently collected specimens deposited in the Fish Collection of the NHMW, Museo Nacional de Ciencias Naturales, Madrid (MNCN), and the Croatian Biology Research Society (HDBI), were included:

*Phoxinellus alepidotus*, two samples from Šatorsko Lake (introduced)—HDBI 261 (2), SL (standard length) 38.5–39.9 mm, coll. D. Jelić, 2013; HDBI 1219 (7), SL 45.6–63.6 mm, collected (coll.) D. Jelić, August 22, 2010.

**FIGURE 2:**
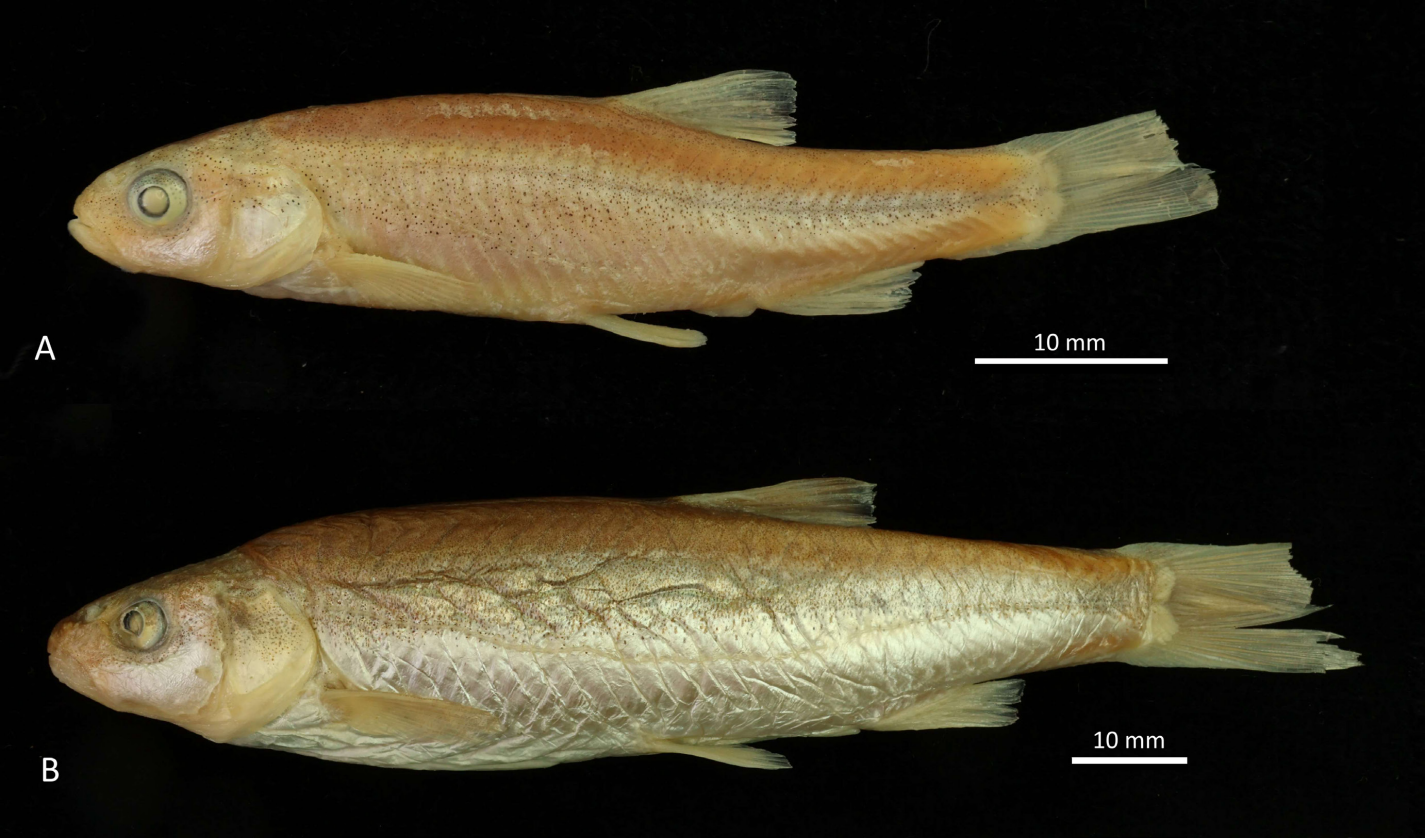
(A) Historical specimen of *Phoxinellus dalmaticus*, NMW 51053:2, SL 49.6 mm, Čikola, 1879. (B) Holotype of *P. pseudalepidotus* , NMW 51102:1, SL 101.8 mm, Mostarsko Blato, 1896.

*Phoxinellus dalmaticus—*NMW (abbreviations for Fish Collection inventory number) 51053 (2), SL 46.8–49.8 mm, Čikola, coll. Kolombatović, 1897 (specimen NMW 51053:2 is photographed in Figure 2A); MNCN 295625 (16), Vrba at Kljake; August 23, 2000; MNCN 293966 (4), Vrba at Kljake; May 20, 2002; MNCN 293238 (5), Vrba at Čavoglave; November 12, 2009; MNCN 295423 (4), same sampling site, May 2, 2002; MNCN 295650 (5), same sampling site, May 17, 2002; all MNCN samples collected by Primož Zupančič.

*Phoxinellus pseudalepidotus*, two samples from Mostarsko Blato—HDBI 1313 (3), SL 55.3–67.9 mm, coll. D. Jelić, 2008; HDBI 1310 (2), SL 40.7–41.6 mm, coll. D. Jelić, September 2008. Holotype of *P. pseudalepidotus* (NMW 51102:1) is photographed in Figure 2B.

In total, 104 specimens of *P. alepidotus* (including putative syntypes), 26 specimens of *P. dalmaticus* , and 141 specimens of *P. pseudalepidotus* were reexamined for morphological study. Of these, 121 adult specimens in better preservation condition (70 of *P. alepidotus* , 8 of *P. dalmaticus* , and 43 of *P. pseudalepidotus* ) were examined for an extended number of morphometric characters (list of characters and their definitions see Table S3) and used in the combined (morphometric and meristic characters together) statistical analyses to test the degree of discrimination of three species and samples (sampling sites) within *P. alepidotus*.

### 2.7 Morphological Analysis

Morphological analysis was performed in order to infer the inter‐ and intrapopulation structure of the species and populations in the *Phoxinellus* genus (goal II), with particular attention to characters known to be associated with hypogean dwelling (Christiansen 2012; Borowsky 2013; Howarth and Moldovan 2018; goal IV).

All specimens were radiographed. Fin ray counts followed Kottelat and Freyhof (2007). Measurements (point‐to‐point) followed Hubbs and Lagler (1958); all measurements are described in the Table S3. Standard length (SL) is measured from the tip of the upper jaw to the posterior margin of hypurals. Head length (HL) is measured from the most anterior point of the upper jaw to the posterior margin of the gill cover, excluding skin. Morphometric characters for descriptive statistics included a total of 29 direct measurements and 57 proportional measurements and ratios. The numbers of counts and measurements used in statistical analyses with different approaches are given in the text and figure captions. The total number of lateral line scales (either complete or interrupted) includes all pored scales. Cephalic sensory canal terminology follows Illick (1956). Vertebral terminology and counts, when applied to *Phoxinellus*, are as explained in Bogutskaya and Zupančič (2003).

Multivariate data analyses were performed, including (i) cluster analysis (CA; using the complete linkage method with Euclidean distance) to detect a general pattern of relative phenotypic similarity between the three predicted species and samples of *P. alepidotus* grouped by sampling sites; (ii) multidimensional scaling (MDS) as a logical final stage of CA to visualize the degree of similarity (relative distances) between the species; (iii) discriminant function analysis (DFA; synonyms LDA, CVA), less sensitive, than CA, to the number of specimens in groups (Da Silva Ramos and Rickard Liow 2024), to classify the specimens into one of the three predicted groups (species); and (iv) principal component analysis (PCA) for *P. alepidotus* as a variable set of specimens grouped by sampling site explaining variance in data, irrespective of the group. In CA, z transformation was used to analyze variables measured at different scales (Romesburg 2004). In all analyses (except for CA and MDS, which were based on average values), to avoid allometric variability of relative values, standardization of absolute measurements was performed (Elliot, Haskard, and Koslow 1995). Statistical analyses were run using Microsoft Excel, Statistica 6.0 (Statistic for Windows. Statsoft), SPSS Statistics V23.0 (IBM SPSS), and PAST version 4.13 (Hammer 1999–2023).

PCA and CA were performed to infer the intrapopulation structure of *P. alepidotus* (goal II) between four samples of *P. alepidotus* specimens grouped according to their sampling sites: (1) Livanjsko Polje (labeled “Livno” in NHMW), (2) Sinjsko Polje (labeled “Sign” in NMW, modern spelling Sinj), (3) unclear sampling site—either Livno or Sinj as explained next and in (Table S3), and (4) Šatorsko Lake (introduced population). The analysis was performed to test whether there is intraspecific phenotypic structure within *P. alepidotus* , and, if so, whether it is possible to identify Livno specimens against Sinj specimens.

## 3 Results

### 3.1 Notes on *Phoxinellus* Ranges, Sympatric (Fish) Species, Subterranean, and Conservation Implications (Partially Summarized Also in Table 1)

#### 3.1.1 Phoxinellus dalmaticus

Historically (NMW 51053) and in recent years (Zupančič and Bogutskaya 2000; Ćaleta et al. 2015, 2019), *P. dalmaticus* is restricted to the upper reaches of the Čikola River and its tributary Vrba River, which flows through Petrovo Polje toward/into the Krka River (Adriatic Sea drainage basin). However, it has never been recorded in the area of the estuary where the Čikola River discharges into the Krka River, and a record of this species in the Krka itself by Mrakovčić, Mišetić, and Povz (1995) was probably a misidentification.

In addition to *P. dalmaticus* , *Telestes tursky* and *Aulopyge huegelii* are native to this area. Among the introduced species, *L. gibbosus* was observed (personal observations NB, DJ).

*Phoxinellus dalmaticus* was observed in high numbers deep in the cave system in the main spring of Čikola during the dives and population monitoring in 2015 and 2018 (DJ, see Supporting Information for detailed description). Individuals were also observed emerging from two smaller springs of Čikola during population monitoring in 2008 and 2015. The Čikola River dries up almost completely in summer (July, August) and the population is maintained mainly by underground retreat, with part of the population remaining stranded in depressions in the riverbed.

In 2004, the species was assessed by the International Union for Conservation of Nature (IUCN) as critically endangered (CR) under criteria B1ab(ii)+2ab(ii) (Crivelli 2006b). A reassessment has recently been submitted to the IUCN (Jelić and Freyhof 2024, in submission) as follows: *P. dalmaticus* has a restricted range (extent of occurrence [EOO] approximately 615 km^2^), which meets the threshold for the endangered category under criterion B1 (EOO < 5000 km^2^). It occurs at two sites where the extent and quality of the habitat is estimated to be declining. Therefore, this species is reassessed as endangered (EN) under criterion B (B1ab[iii]).

#### 3.1.2 Phoxinellus alepidotus

In Grahovo Polje, where the species was abundant more than 50 years ago (Marić 1980, 1983), specimens of *P. alepidotus* were last observed in 2008 (DJ; see also Table 1).

The last published record of *P. alepidotus* in Glamočko Polje was in 2001 (Zupančič and Bogutskaya 2002), and the latest findings are from 2004 to 2009 (P. Zupančič, personal communication); these specimens were collected/observed in the central part of the polje, which drains into the Pliva–Vrbas–Danube drainage (Black Sea drainage basin).

Duvanjsko Polje was included in the range of *P. alepidotus* by some authors. However, Zupančič and Bogutskaya (2002), based on field surveys and intensive sampling by P. Zupančič, suggest that *P. alepidotus* is probably absent from this polje. In summary, no observations, historical or recent samples, are known. During studies in 2002 and 2003, P. Zupančič did not collect *P. alepidotus* in any sampling site of Livanjsko Polje, but Delić et al. (2005), referring to Marić (1983) (not seen by the authors of this study), reported *P. alepidotus* in this polje. The most recent published records of *P. alepidotus* in the northwest of Livanjsko Polje with voucher specimens are from Perea et al. (2010) and Schönhuth et al. (2018).

*Phoxinellus alepidotus* was last confirmed by P. Zupančič during his field work at a site in the central part of Sinjsko Polje (confirmed in 1999; Zupančič and Bogutskaya 2002), but has not been confirmed since then (DJ). In addition, sites on the northern side of Sinjsko Polje were investigated, and large springs that bring water from Glamočko Polje were dived, but no records of *Phoxinellus* were made. Same goes for small springs and streams in the south of Sinjsko Polje, where no records were made.

In Šatorsko Lake, Delić et al. (2005) reported the collection of more than 40 specimens in August 2003 and 2004, which, according to the same reference, are a consequence of unintentional introduction in the 1970s–1980s; *P. alepidotus* was last observed in this lake by DJ in 2013. It has also been observed by DJ in the upper reaches of the Unac River (Black Sea drainage basin; see also next).

In Sinjsko Polje, *P. alepidotus* was found together with the native *Aulopyge huegelii* and the introduced *Cyprinus carpio* and *Pseudorasbora parva* (Zupančič and Bogutskaya 2002). Aside from *P. alepidotus* , *Telestes ukliva* is native to Sinjsko Polje, but it has never been found in the same water bodies, nor in other poljes of the *P. alepidotus* species range (Livanjsko, Grahovo, Glamočko). The only vertebrate stygobiont of the area, the cave salamander ( *Proteus anguinus* ), was not recorded in any of the known *P. alepidotus* sites.

*Phoxinellus alepidotus* was recorded entering some springs in Grahovo Polje in 2008 (DJ), and the springs of the Unac River (tributary of the Una, Danube, Black Sea drainage basin; DJ, in 2008), where it arrived through an underground connection from Šatorsko Lake (DJ). It was also recorded in 2016 to come out of small springs in Livanjsko Polje (DJ).

In 2004, this species was assessed by the IUCN as endangered (EN) under the criterion B2ab (ii, iii, iv) (Crivelli 2006a). A reassessment has recently been applied to the IUCN (Jelić and Freyhof 2024, in submission) as follows: *P. alepidotus* has a restricted range (EOO approximately 2643 km^2^), which meets the threshold for the endangered category under criterion B1 (EOO < 5000 km^2^). It occurs at four sites where the extent and quality of habitat is estimated to be declining. Therefore, this species is assessed as endangered (EN) under criterion B1ab (iii) at the global and European regional scales. In the EU 27 member states, the EOO is approximately 90 km^2^, which meets the threshold for the critically endangered (CR) category (EOO < 100 km^2^), and it is present at one site where the quality of the habitat is estimated to be declining. It is therefore assessed as critically endangered (CR) under criterion B1ab (iii). Downlisting from this category is not considered appropriate as there is no possibility of genetic exchange with the rest of the global population.

#### 3.1.3 Phoxinellus pseudalepidotus

According to historical material held at NHMW and according to the literature (Vuković and Ivanović 1971; Vuković 1977 (at that time still under the name *P. alepidotus* ); Zupančič 2008) *P. pseudalepidotus* has always been restricted to Mostarsko Blato and its Lištica River (Neretva drainage, Adriatic Sea drainage basin). DJ and DM visited Mostarsko Blato in 2008, and at that time *P. pseudalepidotus* was present in large numbers at eight different springs and small streams along the Lištica River. *Phoxinellus pseudalepidotus* accounted for 95% of the fish caught, the remaining 5% being native *Cobitis hercegoviensis* and introduced *Gambusia holbrooki* , *Oncorhynchus mykiss*, and *Salmo trutta*. In 2017, the sites were revisited and *P. pseudalepidotus* was significantly reduced and even rare at some of the sites. In 2020, DM confirmed that the species was rare throughout the Lištica River, possibly due to high water level fluctuations caused by the Mostarsko Blato hydropower plant. It has been observed that *P. pseudalepidotus* spawns in shallow waters, which can dry up due to the water regulation by the power plant, causing the fish roe to dry up as well. DM also reported the problem of illegal fishing, as *P. pseudalepidotus* , though protected by law, is still considered a delicacy by the local community.

In addition to *P. pseudalepidotus* , the native fish fauna of Mostarsko Blato also includes *Salmo obtusirostris* , *Anguilla* (both reportedly absent for 50 years), *Salmo* cf. *farioides*, and *C. hercegoviensis*. There are also a number of introduced species, such as *Salvelinus fontinalis* , *Oncorhynchus mykiss* , *Ameiurus nebulosus*, *Cyprinus carpio*, and *L. gibbosus*, which have become more common over the years (as observed during three different visits to the site between 2008 and 2020). The latter, *L. gibbosus* , appears to be the most invasive and is gradually displacing *P. pseudalepidotus* from its natural habitat. There are no records of *Proteus anguinus* for Mostarsko Blato, but there are records for the nearby sites, where *P. pseudalepidotus* was not recorded.

There is no evidence that *P. pseudalepidotus* occurs underground, none were observed during the cave dives (DJ). There is no information on cave systems in the upper Lištica tributaries where the species might be expected to occur.

In 2004, *P. pseudalepidotus* was assessed by the IUCN as vulnerable (VU) under criterion D2 (Crivelli 2006c). A reassessment has recently been applied to the IUCN (Jelić and Freyhof 2024, in submission) as follows: *P. pseudalepidotus* has a restricted range (EOO approximately 419 km^2^, area of occupancy (AOO) approximately 40 km^2^), which meets the thresholds for the endangered (EN) category under criteria B1 (EOO < 5000 km^2^) and B2 (AOO < 500 km^2^). It is present at a site where the extent and the quality of habitat is estimated to be declining. Therefore, this species is assessed as endangered (EN) under criteria B (B1ab [iii]+2ab [iii]). EU 27 regional assessment: not recorded.

### 3.2 Notes on the Possible Syntypes of *P. alepidotus*

The type locality for *P. alepidotus* given by Heckel (1843: 1040) is the waters around Livno (“aus den Gewassern um Livno in Bosnien”). The number of specimens is not given.

Later, Heckel and Kner (1857: 215, figure 121) gave a detailed description and a drawing of a specimen, and the range of distribution was given as both Sinj and Livno (“Wir fanden diese Art zuerst in Sign und der Narenta während unser gemeinschaftlichen Reise durch Dalmatie, ausserdem erhielten wir sie nur noch von Livno in Bosnien”—“We found this species first in Sign [town of Sinj] and Narenta [Neretva River] during our common journey through Dalmatia, besides that we only got it from Livno [town] in Bosnia”). For this reason, Heckel's specimens from Sinj were considered as “syntypes” in the NHMW Fish Collection, though the type locality is fixed in the original description as Livno (Heckel 1843: 1040).

Currently, the known syntypes include two specimens deposited in the Museum d'Histoire Naturelle de Neuchâtel (MHNN) and the Senckenberg Forschungsinstitut und Naturmuseum, Frankfurt am Main (SMF), labeled as from Livno: MHNN 1018 (1) Dalmatie [Dalmatia]: Livno; SMF 802 (1) Livno. According to Kottelat (1984), all type specimens of the species described by Heckel deposited in the MHNN have labels handwritten by Heckel himself. The SMF specimen was probably also sent there by Heckel, as the date on the label indicates.

**FIGURE 3:**
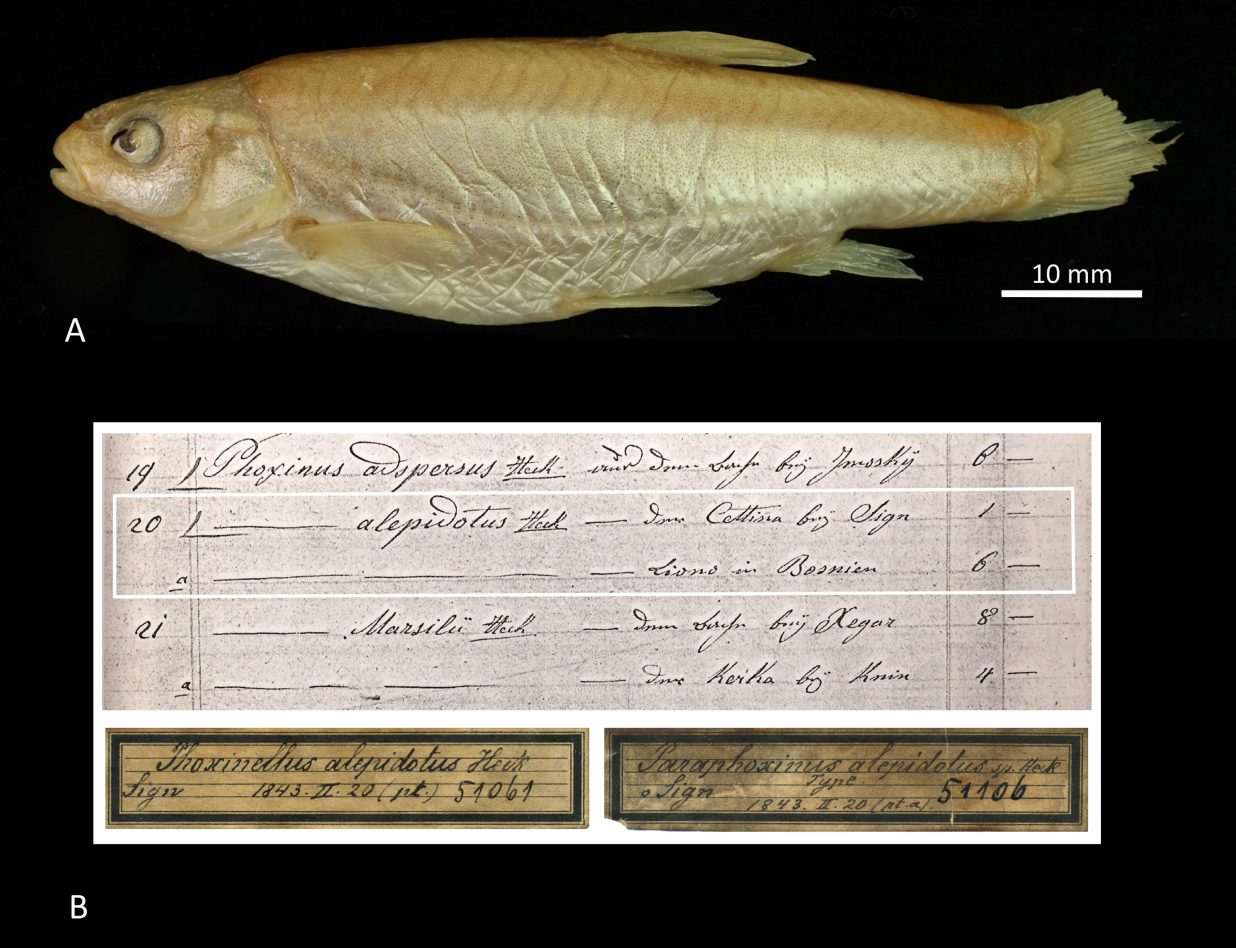
Putative syntypes of *Phoxinellus alepidotus* . (A) *Phoxinellus alepidotus* presumed syntype (see Discussion in the text and Table 3), NMW 51106:2 (Acquisition 1843.II.20 pt. a), SL 70.9 mm. (B) Acquisition entry for 1843.II.20 pt. and for 1843.II.20 pt. a (note the sampling site Livno for 1843.II.20 pt. a) and the old labels.

Concerning the five specimens that are considered to be syntypes of *P. alepidotus* in the NHMW Fish Collection, they are according to the Inventory Book NMW 51061 with three specimens from Sinj with the acquisition entry 1843.II.20 (pt.); and NMW 51106 with two specimens from Sinj with the acquisition entry 1843.II.20 (pt. a) (specimen NMW 51106:2 photographed in Figure 3A). However, according to the Acquisition Book, NMW 51061 lists one specimen from the Sinj sampling site and NMW 51106 lists six specimens from the Livno sampling site (Table 3, Figure 3B). Thus, the sampling site for NMW 51106 may be Livno, corresponding to the type sampling site, and the specimens were mislabeled. However, there is also a discrepancy in the number of specimens between the Inventory Book (labels, jars) and the Acquisition Entry (Table 3). There should be six specimens from Livno, two of which were sent to SNF and MHNN, and four specimens should be in jar NMW 51106 and one (from Sinj) in NMW 51061. However, as the specimens are kept under NMW 51106 (2) and NMW 51061 (3), it is impossible to distinguish between the Sinj and Livno specimens in the NHMW Fish Collection. Finally, DNA analysis of these five specimens was attempted, but was not successful.

**TABLE 3:** Information on putative syntypes of *Phoxinellus alepidotus* from the NHMW Fish Collection. Acqu. no., acquisition number.

Acquisition entry			Inventory book		
Acqu. no.	Sampling site	No. of specimens	Inventory number (NMW)	Sampling site	No. of specimens
1843.II.20 (pt.)	Sinj	1	51061	Sinj	3
1843.II.20 (pt. a)	Livno	6	51106	Sinj	2

### 3.3 Analysis of the Mitochondrial DNA

Of 150 *Phoxinellus* historical specimens collected between 1854 and 1897, at least a part of the COI was successfully amplified and sequenced in 51 (34%) specimens. Of the 51 specimens, a complete mt genome was assembled in eight. Of the remaining 43, a 264‐base pair (bp) long C1 fragment was successfully amplified in eight specimens, a 272‐bp long C2 fragment was successfully amplified in four specimens and a 446‐bp long C1+C2 fragment was successfully amplified and assembled in 31 specimens.

In addition to the eight historical specimens, complete mitochondrial genome was also assembled in seven recent specimens (Table 2), and in three species, *Pe. epiroticus*, *Delminichthys adspersus* and *Telestes polylepis*, representing other missing genera of the subfamily Leusciscinae. The assembled mt genomes are available under GenBank accession numbers PQ431940–PQ431957.

**FIGURE 4:**
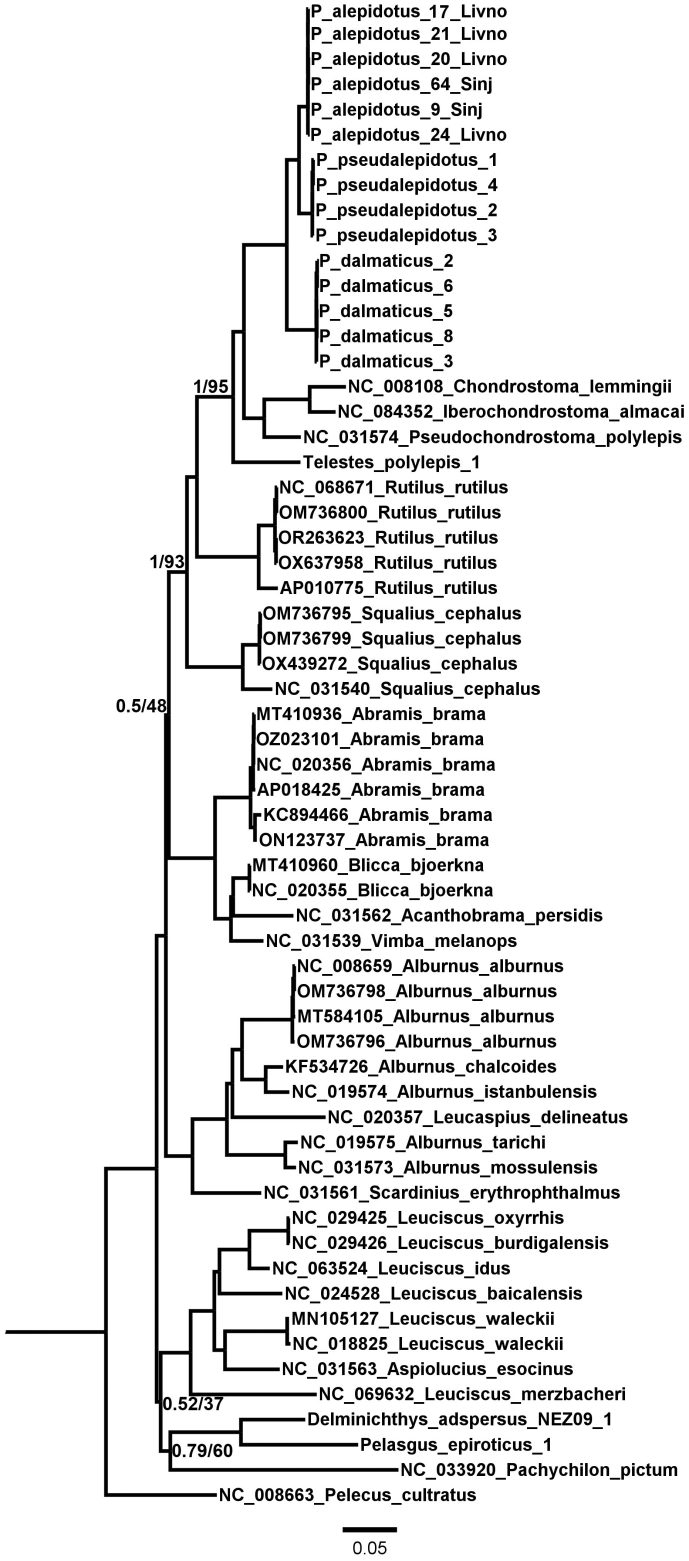
Phylogenetic reconstruction of the subfamily Leuciscinae based on complete mitochondrial genomes using maximum likelihood (software iQTree) and Bayesian inference (software MrBase; both implemented in Phylosuite). *Tinca* (NC_008648) was used as an outgroup (following Schönhuth et al. 2018). Only the nodes with bootstraps/posterior probabilities under 100/1 are shown.

Thus, the alignment was constructed using all Leusciscinae genera (Table S2) and was 16,707‐bp long, including the control region. The general time reversible evolutionary model with proportion of invariable sites and gamma‐distributed rate variation among sites (GTR + I + G) was selected as the best‐fitting model for this dataset. The resulting phylogenetic tree (Figure 4) revealed the sister relationship of *P. alepidotus* and *P. pseudalepidotus* , with *P. dalmaticus* as an outgroup to these two species. The *Chondrostoma* clade was confirmed as the sister group to *Phoxinellus*, and *Telestes* as outgroup to *Phoxinellus*+*Chondrostoma* genera. In general, all relationships were well supported (ML 100, BI 1), except for a polytomy between the groups *Phoxinellus*+*Chondrostoma*+*Telestes*+*Rutilus*+*Squalius* and *Abramis*+*Blicca*+*Acanthobrama*+*Vimba*, and between the groups *Leuciscus*+*Aspiolucius*, and *Delminichthys*, *Pelasgus*, and *Pachychilon*.

The haplotype network was calculated using 55 COI fragments from historical and recent specimens (C1+C2 and complete mt genome), which were 446‐bp long. Out of 39 historical *P. alepidotus* specimens, which were used for the haplotype analysis, 13 were from Livanjsko Polje and 36 were from Sinjsko Polje. All newly generated sequences were deposited in GenBank under accession numbers PQ289565–PQ289608 and PQ315659‐60 (Table 2).

**FIGURE 5:**
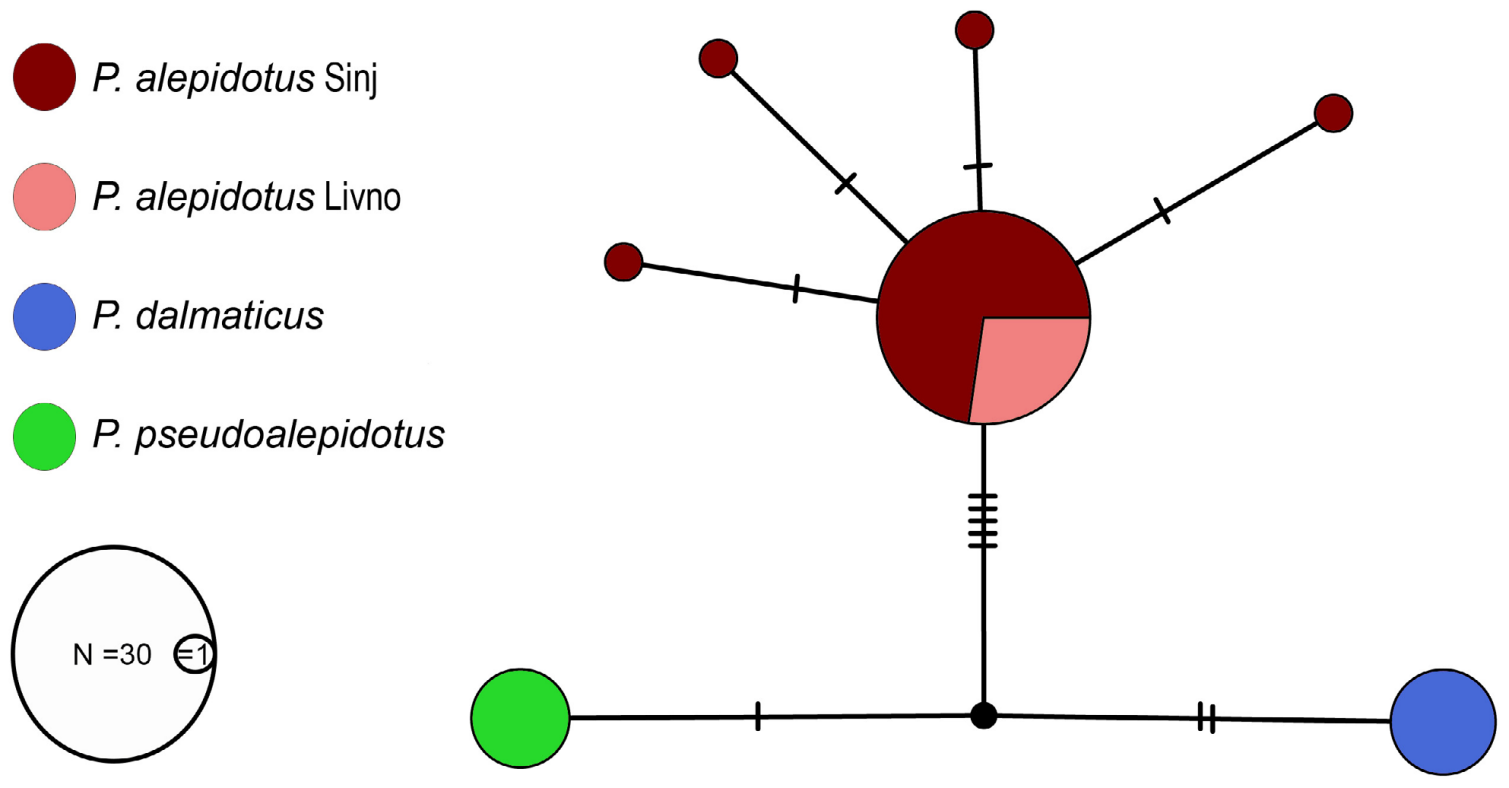
Haplotype network reconstructed in the software PopART v1.7 based on 69 cytochrome oxidase I fragments of 446 base pair of the three *Phoxinellus* species, including recent and historical specimens. Black stripes indicate the number of mutation steps.

The network revealed three distinct groups, corresponding to the three *Phoxinellus* species (Figure 5), with eight mutation steps between *P. dalmaticus* and *P. alepidotus* , and seven between *P. pseudalepidotus* and *P. alepidotus* . There are only four mutation steps between *P. pseudalepidotus* and *P. dalmaticus* . Private haplotypes were detected only in *P. alepidotus* specimens, all four from the Livanjsko Polje. Other specimens (35) clustered to the main haplotype, regardless of their sampling site.

### 3.4 Analysis of the Nuclear DNA

The assembly statistics for Ppse1 indicate a highly fragmented genome with a significant number of small scaffolds (Table S4). The total assembly size is 783.209 MB, with a GC content of 38.12%. The N50 value is 8.036 kb, and the largest scaffold is 85.355 kb. There are 46 scaffolds longer than 50 kb. The scaffold and contig N50 and L50 values, as well as the distribution of scaffold lengths, reflect the high fragmentation of the assembly (Table S4).

The BUSCO (Benchmarking Universal Single‐Copy Orthologs) analysis assessed the completeness of the genomic data and yielded the following results: Of a total of 3640 BUSCO groups searched from the Actinopterygii dataset, 47.2% were identified as complete, 46.3% were single copy, and 0.9% were duplicated. In addition, 13.8% of the BUSCOs were found to be fragmented and 39.0% were missing. These results indicate a moderate level of completeness and fragmentation within the genomic dataset, with a significant proportion of BUSCOs missing (see Table S5 for details). Despite the high level of fragmentation, this assembly provided a valuable resource for subsequent analysis and research.

**FIGURE 6:**
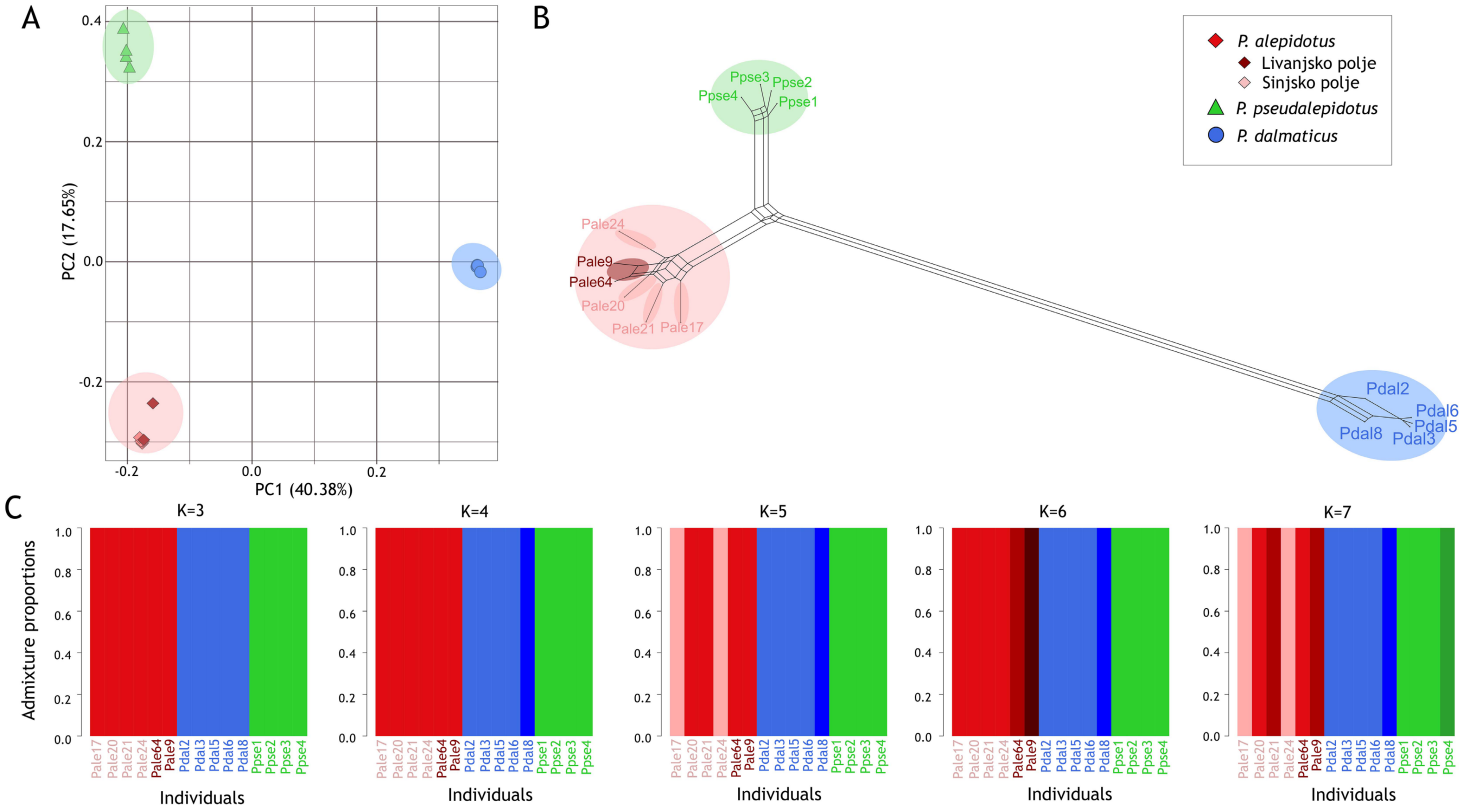
Results of genetic analysis based on 2,715,033 single nucleotide polymorphisms (SNPs) throughout the genomes of *Phoxinellus alepidotus*, *P. pseudalepidotus* , and *P. dalmaticus* acquired with whole genome resequencing on Illumina platform. (A) Principal component analysis (PCA) illustrating the genetic differentiation among the three species: *P. pseudalepidotus* (green), *P. dalmaticus* (blue), and *P. alepidotus* (red). Confidence intervals are given in brackets. (B) Phylogenetic network generated using Splitstree, showing the genetic relationships among the three species. (C) Population structure analysis results from NGSadmix, displaying the genetic composition of the specimens for *K* values ranging from 3 to 7.

Filtering in ANGSD resulted in the identification of 3,972,075 SNPs. A total of 2,715,033 SNPs were used in PCangsd. The PCA results (Figure 6A) showed the presence of three distinct clusters corresponding to the three species: *P. pseudalepidotus* , *P. dalmaticus* , and *P. alepidotus*. Within these clusters, specimens of *P. pseudalepidotus* exhibited the highest degree of individual separation, with the historical holotype specimen Ppse4 positioned farthest from the others. Conversely, specimens of *P. dalmaticus* formed a dense cluster, with only the historical specimen Pdal8 showing slight divergence from the main group. *Phoxinellus alepidotus* specimens also clustered together, but specimen Pale64 from Sinj was separated from the others. Pale64 had a higher coverage (15×) compared to the other *P. alepidotus* specimens (4×–7×), which may explain its distinct position.

The Splitstree network analysis (Figure 6B) also supported the presence of three distinct species. The network showed a clear separation between *P. pseudalepidotus* , *P. dalmaticus* , and *P. alepidotus* , with *P. alepidotus* having the longest genetic distances among the specimens. *Phoxinellus alepidotus* and *P. pseudalepidotus* were found to be more closely related then *P. dalmaticus* .

The NGSadmix analysis was performed for values of *K* ranging from 2 to 7, analyzing 2,715,042 SNPs. At *K* = 3, the three species were clearly separated. When *K* was increased to 4, the historical specimen Pdal8 emerged as distinct. With further increases in *K*, additional substructures became apparent, with the historical specimens of *P. alepidotus* showing the most substructure, but not a stable one and not with a clear separation into two groups according to the Livno and Sinj sampling sites. At *K* = 7, a museum specimen of *P. pseudalepidotus* also showed substructuring.

### 3.5 Morphological Analysis

**FIGURE 7:**
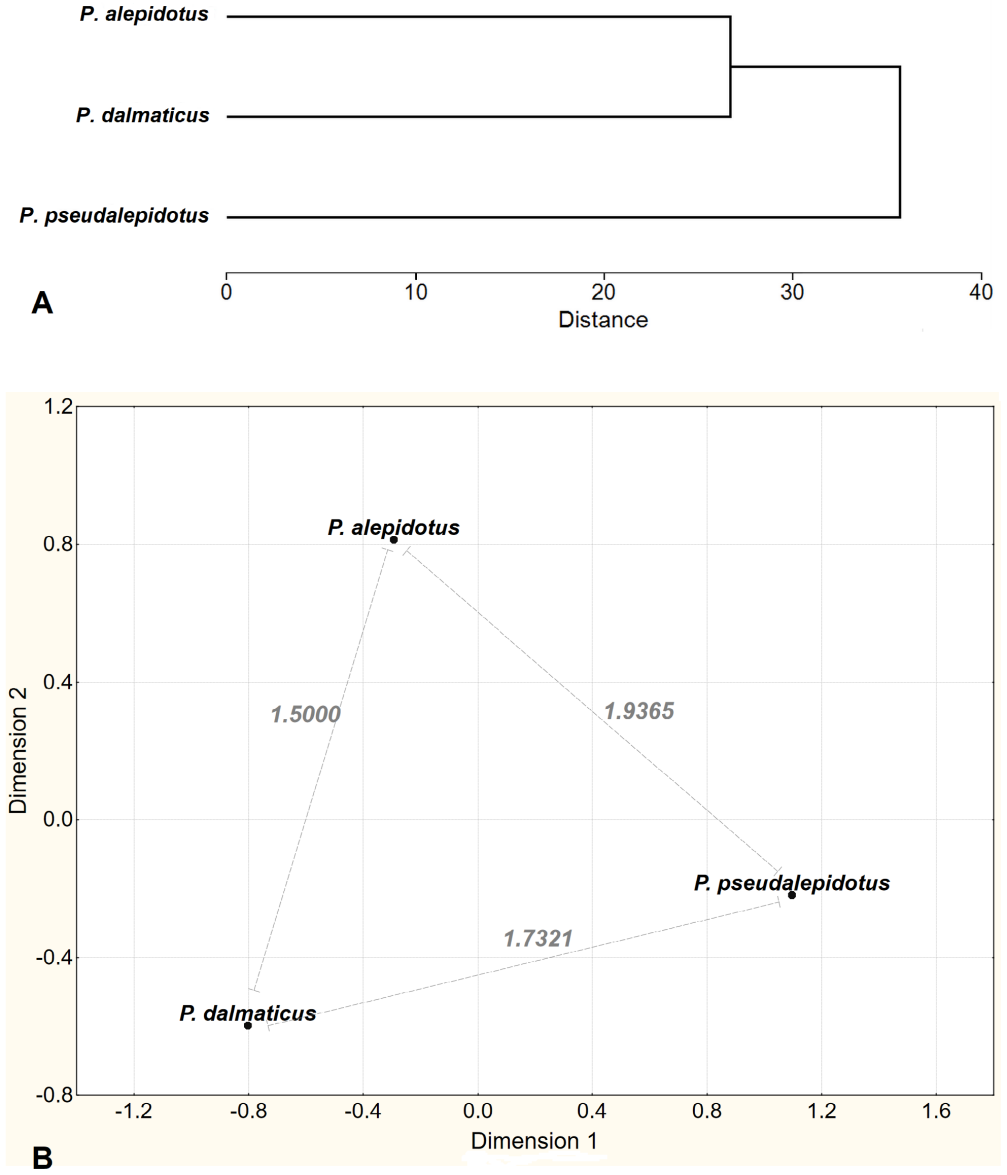
CA (A) and MDS (B) (run in Statistica 6.) based on average values of eight meristic characters and 51 morphometric indexes for three *Phoxinellus* species.

**FIGURE 8:**
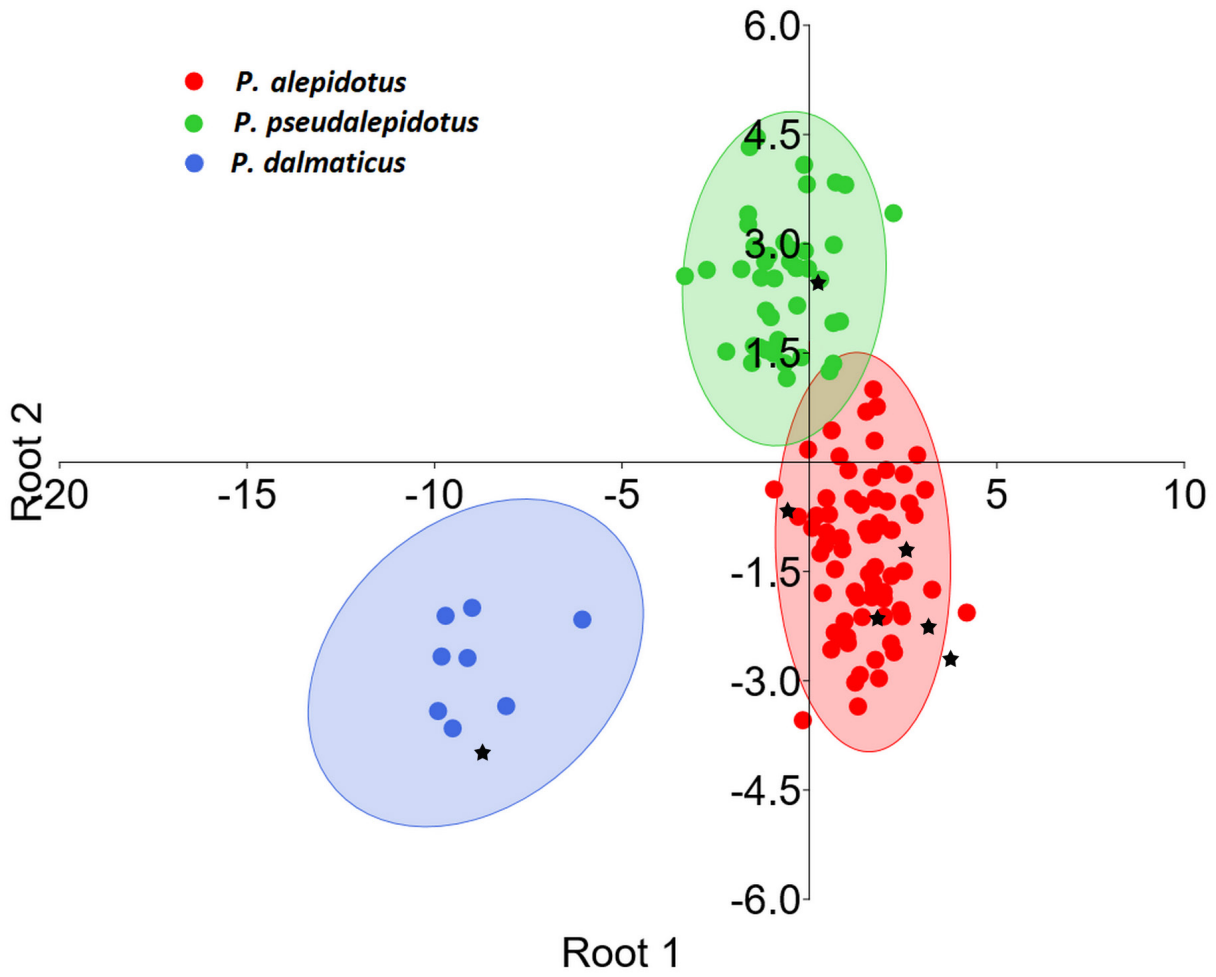
DFA (run in PAST 4.13; 95% confidence ellipses; % of variance 64.4% for root 1 and 35.6% for root 2) based on eight meristic and 28 standardized (according to Elliot, Haskard, and Koslow 1995) absolute measurements for three *Phoxinellus* species. Factor loadings see Table S6. Asterisks refer, respectively, to type specimens: *P. dalmaticus* holotype, CNHM 5387, Cikola River at Kljake, SL 58.3 mm; *P. pseudalepidotus* holotype, NMW 51102:1, SL 101.8 mm; *P. alepidotus* presumed syntypes (see discussion in the text), NMW 51061:1–3 and 51106:1–2, SL 54.6–81.4 mm.

Descriptive statistics per species are given in Table S3. Some distinguishing characters are summarized in Table 4. Morphological analysis of the three *Phoxinellus* species based on CA and MDS (Figure 7) revealed three well‐defined groups corresponding to the three species. In CA, *P. alepidotus* and *P. dalmaticus* were suggested to be the sister species, while *P. pseudalepidotus* was placed as an outgroup to the pair. The three well‐defined species were also observed in DFA, where 100% of the specimens were correctly assigned to each respective group (Figure 8).

**TABLE 4:** Summarized results of morphological analysis. SL, standard length.

Character/Structure	*Phoxinellus pseudalepidotus*	*Phoxinellus alepidotus*	*Phoxinellus dalmaticus*
Size (maximal and average SL)	102.7 mm; 84.5 mm	98.5 mm; 69.8 mm	59.2 mm; 52.1 mm
SL at maturity	48.7 (male), 51.1 mm (female)	43.5 (male), 46.3 mm (female)	42.8 (male), 45.7 (female)
Cephalic sensory canals	Supraorbital and infraorbital canals commonly complete; preoperculo‐mandibular canal only interrupted between angular–articular and preoperculum, present on opercular anterior process	Supraorbital and infraorbital canals commonly fragmented; preoperculo‐mandibular canal interrupted between angular‐articular and preoperculum and commonly absent from opercular anterior process	Supraorbital and infraorbital canals commonly fragmented; preoperculo‐mandibular canal interrupted between angular‐articular and preoperculum and commonly absent from opercular anterior process
Lateral‐line scales	19–73, modally 55–70, mean 58	11–73, modally 20–40, mean 31	16–44, modally 18–30, mean 23
Postcleithrum	Commonly absent	Absent or very small	Absent; rarely present and very small
Scales other than lateral line	Present	Commonly absent	Commonly present
Nuptial tubercles	Absent	Present	Present
Total vertebrae	38–40, modally 39, mean 39	37–41, modally 39–40, mean 39	37–38, modally 37, mean 37
Subterranean status	Presumed basic substygophile due to related species *P. alepidotus* and *P. dalmaticus* and distribution in karst polje, but not yet observed during cave dives	Basic substygophile based on morphology and specimens observed in the caves	Advanced substygophile based on morphology and observations in cave systems

**FIGURE 9:**
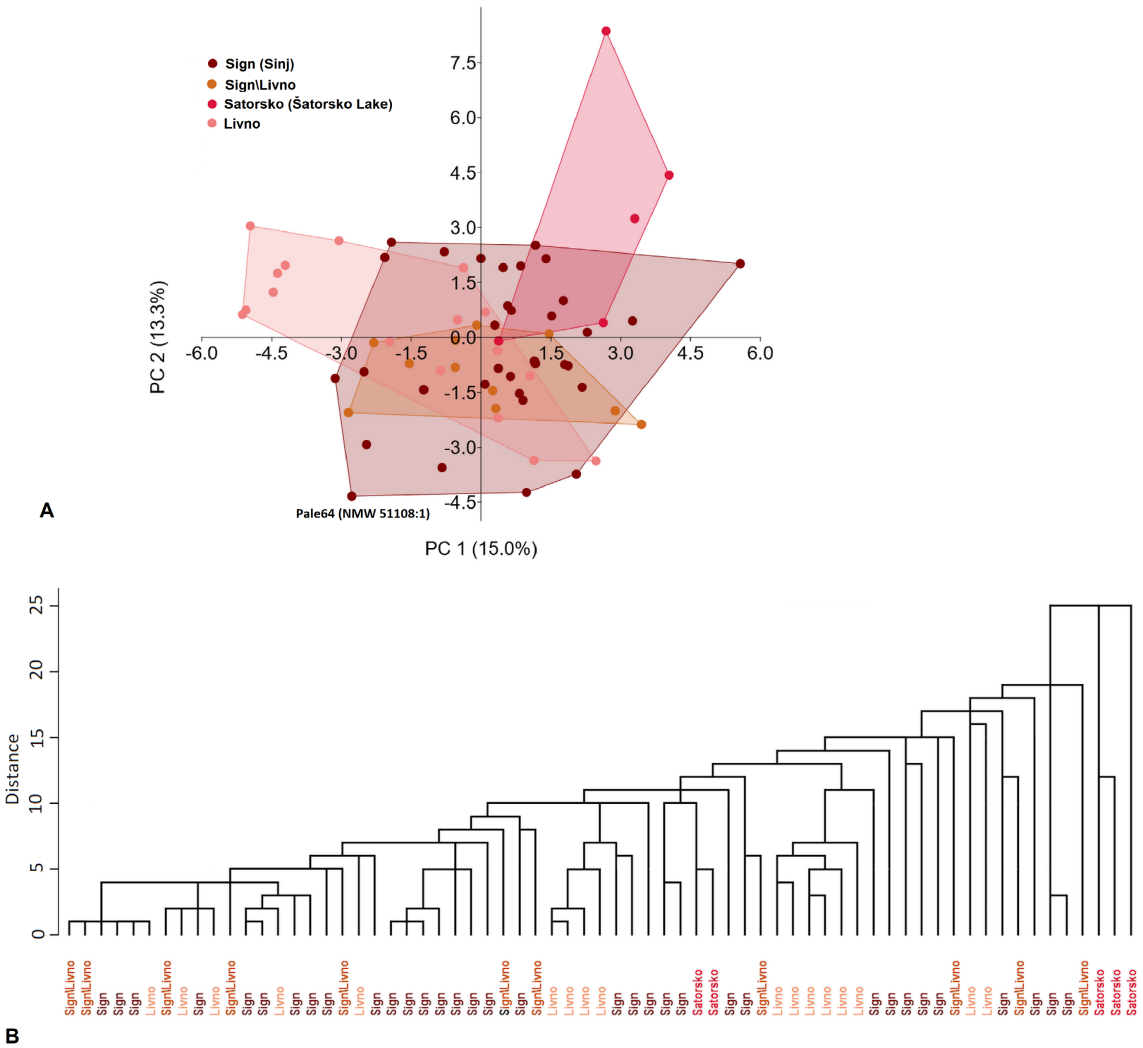
PCA (run in PAST 4.13) and CA (run in SPSS V23.0) using z transformation according to Romesburg (2004), both based on 8 meristic and 28 standardized (according to Elliot, Haskard, and Koslow 1995) absolute measurements for four groups of *Phoxinellus alepidotus* specimens: Collected in Livanjsko Polje (Livno), Sinjsko Polje (Sign), uncertain sampling sites—either Livno or Sinj (Livno/Sign), and Šatorsko Lake (Satorsko; introduced). Factor loadings in PCA see Table S7.

The analysis of *P. alepidotus* samples from different sampling sites revealed that in PCA the first two principal components explain 38% (rather low) of the overall variance (Figure 9A). The mixed distribution of the specimens in the morphospace also demonstrate no intraspecific structure or grouping of the samples by sampling sites (Figure 9A) and does not allow to distinguish between specimens collected in Livanjsko Polje and Sinjsko Polje and, respectively, identify uncertain sampling sites (either Livno or Sinj). The only exception is Šatorsko Lake, which is a bit diverged, but this may be due to its size (very small, lateral line not fully developed) and the small number of specimens examined. The historical specimen Pale64 is found on the margins of the group. In CA, any grouping pattern is also absent (Figure 9B).

## 4 Discussion

### 4.1 Phylogenetic Reconstruction of Leuciscinae Based on Complete Mitochondrial Genomes

As first suggested in Perea et al. (2010) based on COI and cytochrome b sequences, and later in Schönhuth et al. (2018) based on the same two mitochondrial genes as well as a concatenated dataset with two nuclear genes, the phylogenetic relationships within Leuciscinae were confirmed in this study with a phylogenetic reconstruction based on complete mitochondrial genomes. Of particular importance was the provision of complete mitochondrial genomes from all three species of the genus *Phoxinellus* as well as from *Delminichthys*, *Pelasgus*, and *Telestes*, which were previously missing. This study confirmed the sister position of *Phoxinellus* to the *Chondrostoma* clade, with *Telestes* as the sister clade of the pair. In contrast to the phylogenetic reconstructions of previous studies (Perea et al. 2010; Schönhuth et al. 2018), the phylogenetic tree presented in this study (Figure 4) also showed high statistical support for internal nodes, with only three nodes remaining unresolved. The presented phylogenetic reconstruction can also be used for further analyses, especially for the timing of splits between different species and genera, as these analyses were previously based on cytochrome b only and suffered from low statistical support (Perea et al. 2010; Reier, Bogutskaya, and Palandačić 2022).

As in Freyhof et al. (2006), the phylogenetic tree presented here confirms the paraphyletic origin of the three genera *Delminichthys*, *Phoxinellus*, and *Telestes*, and indicates the multiple origins of morphological characters associated with hypogean dwelling, a lifestyle they share. It is widely accepted that most hypogean fishes exhibit a variety of (mostly) reductive modifications associated with cave colonization (Wilkens 2007, 2010), which generally represent analogous (convergent) traits (Christiansen 1961; Wilkens and Strecker 2003; Derkarabetian, Steinmann, and Hedin 2010; Duboué, Keene, and Borowsky 2011; Niemiller et al. 2019). Identifying their closest relatives, in this case the *Chondrostoma* and *Pelasgus* clades, reveals ancestral (plesiomorphic) character states and character transformation within the studied clade (polarity of change) (Desutter‐Grandcolas 1997). Thus, this study suggests a convergent evolution of total body size, lateral line, and scales reduction in *Delminichthys*, *Phoxinellus*, and *Telestes*, which are not closely related. This conclusion coincides with the preliminary hypothesis of Bogutskaya and Zupančič (2003) that the “reduction of scales” found in these three genera originated from different plesiomorphic states and by different types of transformations. There was the complete loss of scales and then the shortening of the lateral line in *Phoxinellus*, in contrast to the presence of scales on the entire body, albeit progressively reduced, nonoverlapping, embedded in the skin, and poorly ossified, together with a comparatively long lateral line in *Delminichthys* and Dinaric *Telestes* species (Freyhof et al. 2006).

### 4.2 Intraspecific and Interspecific Structure in the Genus *Phoxinellus*

According to previous morphological studies (Zupančič and Bogutskaya 2000, 2002; Bogutskaya and Zupančič 2003), the three *Phoxinellus* species are well supported. Furthermore, molecular studies have confirmed their placement in the genus *Phoxinellus*, clearly separated from the species placed in the genera *Delminichthys* and *Telestes* (Freyhof et al. 2006; Perea et al. 2010; Geiger et al. 2014; Schönhuth et al. 2018). In this study, both morphological (Figures 7 and 8) and molecular analyses based on mitochondrial data (Figures 4 and 5) clearly confirmed these findings, which were further supported by molecular analyses based whole genome data (Figure 6). While the molecular analysis of putative *P. alepidotus* syntypes failed, this study provided an additional clarification of their syntype series (Figure 3, Table 3 and section 3.2 in Results). In addition, the holotype of *P. pseudalepidotus* (Figure 2B), collected in 1896, was successfully analyzed, providing a complete mitochondrial genome and nuclear DNA data for the type specimen, providing an explicit link between a genetic lineage and the species name (Prosser et al. 2016; Castañeda‐Rico et al. 2022).

When looking at the population structure within each *Phoxinellus* species, the number of specimens analyzed was too small to provide clear results. Ideally, 10–15 specimens from both recent and historical collections would be analyzed in parallel to provide insight into population structure and its change over time. However, this was not possible herein because recent specimens are difficult to collect due to their declining abundance. For historical specimens, DNA fragmentation coupled with low quantity is typical (Hagelberg, Hofreiter, and Keyser 2015; Hawkins et al. 2022). Thus, of the 150 historical specimens analyzed, 34% were successfully amplified for at least a short COI fragment, which is consistent with the success rate previously reported for the NHMW Fish Collection (Palandačić et al. 2020). This percentage was sufficient to clearly delimit between the three *Phoxinellus* species, but provided little insight into the population structure of each species.

Despite of the low number of specimens included in the molecular analysis, some basic conclusions about the intrapopulation structure can be drawn. For example, the haplotype network including 13 *P. alepidotus* from Livanjsko Polje and 36 from Sinjsko Polje showed no difference between these two sampling sites (Figure 5). Morphological analysis, including samples from all four karst poljes with confirmed occurrence of *P. alepidotus* as well as the introduced population from Šatorsko Lake, also rejected the differentiation of the samples based on their sampling sites (Figure 9). Population structure analysis of nuclear data using ANGSD software was inconclusive due to the low number of specimens, that is, two specimens from Sinjsko and four from Livanjsko Polje (Figure 6C). However, it seemed to confirm that the specimens belonged to one population. Taken together, these findings suggest gene flow between different poljes. The lower part of Grahovo Polje, Livanjsko Polje, and Sinjsko Polje belong to the Cetina River drainage (Adriatic Sea drainage basin) and are connected by surface and underground water links (Baučić 1967; Magdalenić 1971; Štambuk‐Giljanović 2002; Bonacci et al. 2020; see also Figure 1). Thus, it is possible that this complex water system hosts a single interconnected population of *P. alepidotus* , as suggested by a population study of *Delminichthys adspersus* (Palandačić, Zupančič, and Snoj 2010), which has a similar, partially hypogean (Jelić, Špelić, and Žutinić 2016) lifestyle. However, it is possible that the lack of differences between populations is due to their recent divergence (2.6 mya for split of the three *Phoxinellus* species; Perea et al. 2010; Reier, Bogutskaya, and Palandačić 2022) rather than ongoing gene flow, as suggested by a recent study of *Phoxinus lumaireul* in the northwestern Dinaric Karst (Reier et al. 2024 in submission), but again the dataset is too small to draw firm conclusions.

In the population structure analysis of *P. dalmaticus* and *P. pseudalepidotus* , historical specimens of both species were labeled as distinct (Figure 6C). As they were collected more than 100 years apart, this could be due to genetic drift typical for small populations (e.g., Coleman et al. 2018) with a relatively short generation time (approximately 2–4 years according to field surveys, but not confirmed in a scientific study). However, it could also be due to differences in the quality of the historical versus recent DNA, leading to artifacts in the downstream analysis. This is seen in the example of specimen Pale64, which had a much higher genome coverage than the rest of the Pale specimens, and it also appears different in the PCA analysis. Yet, this specimen was also identified as slightly different in the PCA analysis (Figure 9A), where it is placed at the edge of the group.

### 4.3 Notes on the Current Species Distribution Areas and Conservation Status

All three *Phoxinellus* species have restricted ranges, with *P. dalmaticus* and *P. pseudalepidotus* each occurring in only one karst polje, while *P. alepidotus* was (historically) present in four, as no confirmed sightings are known for Duvanjsko Polje (Table 1). However, some of the sites with thriving populations of *P. pseudalepidotus* in 2008 are now devoid of fish (confirmed by monitoring in 2020, see Section 3 and 3.1), whose decline was probably caused by the construction of the hydropower plant (HPP Mostarsko Blato) in 2010. Its reservoir is subject to significant water level fluctuations. *Phoxinellus pseudalepidotus* spawns in shallow parts of the lake and in irrigation canals, where drops in the water level can cause the roes to dry out. *Phoxinellus dalmaticus* is present in good numbers in the Čikola River (Petrovo Polje), but due to increased water use and climate change, the river is exposed to prolonged draughts (Crivelli 2006b).

As for *P. alepidotus* , it was last recorded in Sinjsko Polje during the study by Bogutskaya and Zupančič in 2002, but was never confirmed by DJ during many subsequent monitoring events, and is therefore considered extirpated by the authors of this study. Similarly, the last recorded observation of *P. alepidotus* in Glamočko Polje was in 2009 (by P. Zupančič, personal communication), but as there is no knowledge of subsequent monitoring studies, this polje is retained in the current distribution area of *P. alepidotus* . On the other hand, *P. alepidotus* was introduced into Šatorsko Lake and spread to the upper parts of the Unac River, possibly through the underground water connections.

Introduced fish species are another cause for concern. Jelić, Špelić, and Žutinić (2016) reported serious impacts of non‐native fish species on endemic fishes in the Lika region, including two *Delminichthys* species. Similar to *Phoxinellus*, they retreat underground during draughts, and perhaps also to escape introduced predators. In Mostarsko Blato, at least five non‐native fish species have been reported that are known to prey on *P. pseudalepidotus* and/or increase eutrophication in the naturally oligotrophic ecosystem. For the Čikola River (Petrovo Polje), *Gambusia holbrooki* was reported (Andabaka, Senta, and Gudelj 2021) and *L. gibbosus* was observed during this study. Thus, all three species are endangered and need to be closely monitored in the coming years.

### 4.4 Notes on Hypogean and Ecological Status of *Phoxinellus*

There are many different classifications and criteria applied to subterranean biota (for reviews on this topic, see e.g., Sket 2008; Trajano 2012; Trajano and de Carvalho 2017; Culver and Pipan 2019). As explained in the Introduction, the three *Phoxinellus* species of this study undoubtedly belong to the category of substygophiles (for a detailed discussion of the history of the term, see Sket (2008: pp. 1558–1559)), defined on the basis of the need to utilize the surface environment for at least one vital function (in their case, spawning). It is important to recognize that the periodicity to which substygophiles are subjected can vary over a wide range, from diurnal to once in several years. Thus, Jelić, Špelić, and Žutinić (2016), at the point still calling them stygophiles, depending on the duration of the species occurrence in hypogean conditions, established subcategories as “basic stygophiles” and “advanced stygophiles”, which are reclassified here as “basic substygophiles” and “advanced substygophiles”, However, in contrast to Jelic et al. (2016), the subcategories of substygophiles in this study were determined based on (confirmed) observations in caves as well as morphological traits (described in detail next). *Phoxinellus dalmaticus* is classified as an advanced substygophile, and *P. alepidotus* and *P. pseudalepidotus* , which were never observed in cave systems but occur in springs ( *P. alepidotus* ) and are commonly described by locals as “being thrown out of the springs by the water” (Ćurčić 1913, 1916), are classified as basic substygophiles. Yet, according to the morphological analysis, the latter two also show different levels of adaptation to subterranean life (see next).

*Phoxinellus*, similar to *Telestes* and *Delminichthys*, do not demonstrate any true troglomorphism, or troglomorphosis, for example, a pale coloration, the reduction or loss of eyes (Wilkens 2010; Niemiller et al. 2019), as they obligately spend, more or less regularly (depending on environmental conditions), at least a short time outside subterranean environments for spawning. Spawning per se was never observed by the authors of this study, but males and females with gonads in the latest stage of maturation were always found only outside caves. The historical NHMW samples containing mature adults also appear to have been taken from surface waters, as not a single record indicates a “cave” site. Thus, the phenomenon of substygophily is a good example of the trade‐off when, within an organism lifetime, it has a limited amount of energy/resources available during its lifetime and must constantly divide it between different functions (Garland 2014; Garland, Downs, and Ives 2022), such as adapting to the hypogean aquatic environment during the lack of surface waters and spawning outside the caves when the water is available.

In accordance, some morphological features modified in cave fishes are present in *Phoxinellus*. They lack scales on the body (when present, then only a few) except on the lateral line. The lateral line is reduced to varying degrees (see Table 4 for details). *Phoxinellus dalmaticus* has the most significantly reduced (shortened and interrupted) lateral line and a comparatively more fragmented cephalic sensory canal system, in accordance with its advanced substygophile status. Of the three species, *P. pseudalepidotus* , a basic substygophile that has never been observed during cave dives, has the longest lateral line and an almost complete cephalic sensory system. *Phoxinellus alepidotus*, a basic substygophile, demonstrates an intermediate state with moderate reduction of the lateral line and the cephalic sensory system. The degree of morphological changes that may be induced by their different adaptations to hypogean life is also reflected in the morphological analysis, where the CA analysis (Figure 7A), in contrast to the molecular phylogenetic reconstruction (Figure 4), and previous studies (Perea et al. 2010; Schönhuth et al. 2018), suggests a closer phenotypic relationship between *P. dalmaticus* and *P. alepidotus* , while *P. pseudalepidots* was placed as an outgroup to the pair. This relationship may reflect the degree of their specialization, that is, the morphological traits indicating adaptation to the subterranean environment, and is not entirely consistent with the findings of Lukač et al. (2023), who suggested that body shape in Dinaric fishes could be used as an indicator of phylogenetic relationships.

In contrast to *Delminichthys* and *Telestes*, which occur in sympatry (Mrakovčić et al. 2006; Jelić, Špelić, and Žutinić 2016), *T. ukliva*, though reported for Sinjsko Polje, does not co‐occur with *P. alepidotus* , a fact that may indicate their trophic competition. This agrees with Mrakovčić et al. (2006), who reported that they both feed on small invertebrates and their larvae. However, the reason could also be the different water type preferences of the two species, as *T. ukliva* prefers clear, fast‐flowing water while the two lakes, where *P. alepidotus* was found, are stagnant (personal observation of DJ).

*Phoxinellus* species were also not found to coexist with *Proteus anguinus* (observation by DJ, but see also Bizjak Mali and Sket 2019), for example with *P. dalmaticus* , which is present in the Čikola springs and nearby caves, but not in the cave systems where Čikola flows into the Krka River and cave systems connected to the Krka River, known sites inhabited by *P. anguinus* . In addition, *P. anguinus* has never been reported in Mostarsko Blato. According to Recknagel et al. (2022; and references therein), the main diet of *P. anguinus* in the caves is crustaceans such as *Troglocaris* and *Niphargus* (Aljančič 1961; Briegleb 1962; Delić and Fišer 2019). Yet, it has been suggested that *P. anguinus* migrates to karst springs where there is a higher density and diversity of available prey, and where, according to Bressi, Aljančič, and Lapini (1999), they prey on oligochaetes and tadpoles. Bressi (2004) and Balázs and Lewarne (2017) also reported a single case of *P. anguinus* ingesting a minnow (*Phoxinus* sp.), so it is also be possible that *P. anguinus* preys on the juvenile *Phoxinellus*. It is currently unknown how often *P. anguinus* accesses springs (Recknagel et al. 2022), but its predatory behavior may explain the mutual competitive exclusion between *P. anguinus* and *Phoxinellus*, at least, during early life stages.

### 4.5 Influence of the Paleohydrology on the Distribution of *Phoxinellus*

Rather than ecological exclusion, the differences in the distribution of fishes and *P. anguinus* could be due to hydrogeological events in this area. For example, the ichthyofauna of the Čikola River is composed of different species than the ichthyofauna of the Krka River, suggesting their independent development, regardless of their current hydrological connection. In addition to *P. dalmaticus* , the endemic *Telestes tursky* and *Aulopyge huegelii* are found in the Čikola River system and are absent from the rest of the Krka River drainage. The only report of *Phoxinellus* from Krka, mentioned in Mrakovčić, Mišetić, and Povz (1995), was never confirmed, and seems to be a misidentification. Fish species composition in the Čikola River is much more similar to the Cetina River, with phylogenetically close *Phoxinellus* ( *P. alepidotus* ) and *Telestes* (*T. ukliva*).

This pattern of species distribution seems to be reflected in the hydrogeological history of the area. Namely, during the early and middle Miocene, a series of extensive intramontane lake basins formed in the area of the Dinaric Mountains, probably as a result of extensional tectonics (de Leeuw et al. 2012). These basins covered much larger areas than the present‐day karst poljes, which were then part of unified basins (e.g., Vrlika‐Sinj‐Drniš Basin and Livano‐Duvno Basin). Given the predominance of carbonate bedrock, it is likely that these basins were also connected by underground karst streams. In accordance, research on Miocene lake sediments have shown that the area of Petrovo Polje (with the Čikola River) was part of the same large lacustrine depositional system as Sinjsko Polje (with the Cetina River) during the early and middle Miocene (Neubauer, Mandic, and Harzhauser 2015). At the end of the Miocene, as a result of the inversion from extensional to compressional tectonics in the Dinarides, the gradual disintegration of this large basin into smaller, mutually separated basins (today's karst poljes) began (de Leeuw et al. 2012; van Unen et al. 2019). Although the disintegration process is assumed to have started in the late Miocene or early Pliocene, this process has continued gradually until the present day, with the reshaping of the river drainages occurring over time. Thus, it is possible that the final splits between the closely related fish species *P. alepidotus* / *P. dalmaticus* and *T. ukliva*/*T. tursky* happened in the middle/end of the Pliocene (Perea et al. 2010; Reier, Bogutskaya, and Palandačić 2022), especially since the divergence time estimates have wide intervals and need to be considered with caution (Reier, Bogutskaya, and Palandačić 2022). The separation of the Čikola River from the Cetina River and the present connection of the Čikola River with the Krka River via the canyon part of the Čikola River's watercourse (with intermittent flow) probably happened much later, during the Pleistocene.

## 5 Conclusion

*Phoxinellus* minnows have adapted to the highly variable water conditions of karst poljes, ranging from floods to draughts within shorter (seasonal, annual) and longer (decadal) periods. Thus, they have adapted to fluctuating water conditions and have been quoted as spending “up to several months underground.” However, in this study, based on the analysis of their morphological characters and their presence (or absence) in caves, they were classified more precisely, with *P. dalmaticus* as an advanced substygophile and *P. alepidotus* and *P. pseudalepidotus* as basic substygophiles. The introduced/invasive species threatening *Phoxinellus* populations were identified. Since the reduction of their population size and distribution was also documented herein, studies reporting on their ecology are even more important. In this study, an integral approach of molecular and morphological analyses coupled with field surveys and paleohydrology helped to elucidate different aspects of the biology of these locally endemic species, which will be crucial for their conservation. Finally, with the use of historical collections, we were able to include extirpated populations of *P. alepidotus* in the analysis.

## Author Contributions

**Anja Palandačić:** conceptualization (equal), data curation (equal), formal analysis (equal), funding acquisition (equal), project administration (equal), resources (equal), software (equal), supervision (equal), validation (equal), visualization (equal), writing – original draft (equal), writing – review and editing (equal). **Susanne Reier:** data curation (equal), formal analysis (equal), resources (equal), software (equal), visualization (equal). **Oleg A. Diripasko:** formal analysis (equal), methodology (equal), visualization (equal), writing – review and editing (equal). **Dušan Jelić:** data curation (equal), formal analysis (equal), resources (equal), writing – review and editing (equal). **Alexandra Wanka:** formal analysis (equal), resources (equal), writing – review and editing (equal). **Andrej Stroj:** visualization (equal), writing – review and editing (equal). **Dario Marić:** data curation (equal), resources (equal), writing – review and editing (equal). **Nina G. Bogutskaya:** conceptualization (equal), data curation (equal), formal analysis (equal), investigation (equal), resources (equal), supervision (equal), visualization (equal), writing – original draft (equal), writing – review and editing (equal).

## Conflicts of Interest

The authors declare no conflicts of interest.

## Supporting information


Table S1‐S7.

## Data Availability

All newly acquired molecular sequences are available in GenBank under the accession numbers PQ289565–PQ289608 and PQ315659‐60 (COI) and PQ431940–PQ431957 (complete mitochondrial sequences). Sequences resulting from the whole genome sequences are available at NCBI under BioProject PRJNA1168679 (individual accession numbers SAMN44055077‐91, see Table 2). Morphological data for statistical analyses are available in Supporting Information (SI1–SI7).
