# Supplementary material for: Substygophily in Dinaric Karst: A Model Case of Locally Endemic Minnows Phoxinellus (Leuciscinae)
Source: Ecol Evol. 2024 Dec 23;14(12):e70648. doi: 10.1002/ece3.70648 (PMC11664320; doi:10.1002/ece3.70648)
Supplement: Supplementary file 1 — Table S1‐S7. [file ECE3-14-e70648-s001.docx]

***Supplementary material to Substygophily in Dinaric Karst: a model case of locally endemic minnows Phoxinellus (Leuciscinae)***

Anja Palandačić, Susanne Reier, Oleg A. Diripasko, Dušan Jelić , Andrej Stroj, Alexandra Wanka, Dario Marić, Nina G. Bogutskaya

**2. Materials and Methods**

**2.1 Description of species distribution areas**

(Additional data in Supplementary Table S1)

*P. dalmaticus* range

The species range of *P. dalmaticus* is Petrovo Polje with the main course of the Čikola River (Bonacci et al. 2018, Andabaka et al. 2021). Čikola has a total length of 46 km and is a combination of a flat course through Petrovo Polje, while the last 14 km it flows through a deeply cut canyon into the Krka River near Torak Lake. Although it is almost dry in the summer, in the winter months, especially after heavy rains, it flows along its entire length. Torak is actually a karst pit with a diameter of 150 m and a depth of 47 m, from which the water springs. The water regime of Petrovo Polje was first influenced by a water pumping station at the Torak spring (started in 1955 with a capacity of 50 l/s), from which the town of Drniš received water. This station is currently out of order, but the pumping station buildings are still maintained. At the end of the 1980s, another water pumping station was built at the main source of the Čikola River with a capacity of 180 l/s. In general, this would be about 5-8% of the source capacity on average (Bonacci et al. 2018). The water is extracted through pipes that have been borrowed into the cave system at about 50 meters from the source. These are visible inside the cave. Water extraction has no visible effect on the regularity of flooding.

*P. alepidotus* range

Present distribution of *P. alepidotus* includes four karst poljes. One additional polje is mentioned in the literature, while the species has also been introduced into a lake outside its native range.

Grahovo Polje has one main river, the Korana, which flows into a tributary of Cetina River (in Livanjsko Polje, Adriatic Sea drainage basin). However according to Bonacci and Ljubenkov (2005), Korana drains mainly into the upper Krka River (also Adriatic Sea drainage basin). However, according to Delić et al. (2005), the upper parts of Grahovo Polje drain towards north-east and the water possibly flows to Una River (Danube, Black Sea drainage basin) (Marić 1980). Near Grahovo Polje, there is also Šatorsko Lake, where *P. alepidotus* was introduced (Delić et al. 2005). This water body belongs to the Black Sea drainage basin; it flows into the Unac River, a tributary of the Una River (Sava-Danube – Black Sea drainage basin). Grahovo Polje is connected to Livanjsko Polje by a small stream, which flows through a narrow corridor between the slopes of mountains (Roglić 1954, Štambuk-Giljanović 2002).

Glamočko Polje with several small watercourses is hydrologically divided into three parts. The most southern part drains to Cetina River and thus Adriatic Sea drainage basin, while the central and the northern parts drains to Pliva River (tributary to Vrbas, Danube) and to Sana (tributary to Una, Danube), respectively, and thus to Black Sea drainage basin.

Duvanjsko Polje is hydrologically a part of the Cetina River drainage, with the Šujica River as its main stream. Duvanjsko Polje was mentioned as part of the species range of *P. alepidotus* in Ćurčić (1916), Taler (1953), Sabioncello (1967), Vuković & Ivanović (1971), Vuković (1977, 1982). The waters drain underground to Livanjsko Polje.

The main stream of Livanjsko Polje is Bistrica, which receives its water from many small streams in the polje. It flows unregulated for about 6.3 kilometers through Livanjsko Polje before it reaches the section from where the rest of its course flows regulated through artificially created riverbed, which is simply dug into the ground. After 14.9 kilometers, the Bistrica receives a significant amount of water from the northwestern half of Livanjsko Polje, which is channeled through an artificial canal. At 20.3 km, the Bistrica reaches the first and smaller of two artificial reservoirs for the hydroelectric power plant, the other being the Buško Blato reservoir. Buško Blato (the south-eastern part of Livanjsko Polje) was essentially agricultural land until 1974, when it was converted into an artificial reservoir. Lake Blidinje is another water body in the south of Livanjsko Polje, which is a product of anthropogenic intervention and activities of human inhabitants (created at the end of the 19th century in order to preserve karst water), and is mentioned by Ćurčić (1916), Taler (1953), Sabioncello (1967), Vuković and Ivanović (1971), Vuković (1977, 1982) as a locality with the occurrence of *P. alepidotus*.

Sinjsko Polje with its main stream Cetina drains into the Adriatic Sea. Only two exact localities, small karst lakes Stipančevo and Miloševo are known. It cannot be excluded that historically *P. alepidotus* inhabited all six poljes that existed along the Cetina valley (Cetinsko, Vrličko, Koljansko, Ribarićko, and Sinjsko) (Baučić 1967), now covered from Vrlika down to Livanjsko Polje by the artificial lake Peruća (Perućko Lake) (Cetina was dammed by the Peruća Hydroelectric Power Plant constructed in 1958); historical locality Sign (Sinj) can possibly indicate not only Sinjsko Polje but the entire area adjacent to the town of Sign (the Cetina Valley). Historically, Sinjsko Poljes was periodically flooded but never at present as it is regulated by the Peruća dam.

*P. pseudalepidotus* range

The species range of *P. pseudalepidotus* is Mostarsko Blato (defined as Mostarsko Blato subcatchment b in Filipović et al. 2023) with the Lištica River draining system. Out of 42.56 km^2^, 38 km^2^ are exposed to seasonal flooding. During the rainy season from October to June, considerable amounts of water enter the polje. The water comes from the surrounding slopes or from karst springs and estavelles on the edges of the polje, while part of the water also comes from the sinking rivers. This creates lakes of varying size and duration. Most water from the polje flows underground to the Neretva River (Miličević 2009, Filipović et al. 2023). Since 2010, the water regime of the polje is influenced by the hydroelectric power plant, for which two artificial canals and two artificial lakes were created.

**Table S1a: *P. dalmaticus* & *P. pseudoalepidotus***

| **Source** | **ScientificName** | **Native Status** | **Country** | **Basin** | **Drainage** | **Polje** | **Locality** | **Latitude decimal** | **Longitude decimal** | **Date** |
| --- | --- | --- | --- | --- | --- | --- | --- | --- | --- | --- |
| Zupancic & Bogutskaya (2000) | *Phoxinellus dalmaticus* | Native | CROATIA | Adriatic Sea | Krka | Petrovo Polje (upprt part as Mirlović Polje) | Vrba creek (Čikola upper reach) at Kljake | 43,79098 | 16,31473 | 1998 |
| Zupancic & Bogutskaya (2000); Geiger et al. (2014) [Fischsammlung Jörg Freyhof, Berlin, Germany] | *Phoxinellus dalmaticus* | Native | CROATIA | Adriatic Sea | Krka | Petrovo Polje (upprt part as Mirlović Polje) | Vrba creek (Čikola upper reach) at Kljake, 2 | 43,79112 | 16,314633 | 2000 |
| PZ collection | *Phoxinellus dalmaticus* | Native | CROATIA | Adriatic Sea | Krka | Petrovo Polje (upprt part as Mirlović Polje) | Vrba creek (Čikola upper reach) at Čavoglave | 43,78071 | 16,32678 | 12 Nov 2009 |
| Zupancic & Bogutskaya (2000) | *Phoxinellus dalmaticus* | Native | CROATIA | Adriatic Sea | Krka | Petrovo Polje (upprt part as Mirlović Polje) | Čikola River at Ružič | 43,81719 | 16,26775 | 1996 |
| Zupancic & Bogutskaya (2000) | *Phoxinellus dalmaticus* | Native | CROATIA | Adriatic Sea | Krka | Petrovo Polje (upprt part as Mirlović Polje) | Čikola River north of Kljake | 43,80477 | 16,31279 | 21.08.1998 |
| NMW 51053 | *Phoxinellus dalmaticus* | Native | CROATIA | Adriatic Sea | Krka | Petrovo Polje (upprt part as Mirlović Polje) | Čikola River |  |  | 1897 |
| DJ 2024 (unpublished data) | *Phoxinellus dalmaticus* | Native | CROATIA | Adriatic Sea | Krka | Petrovo Polje (upprt part as Mirlović Polje) | Čikola spring (main) | 43,79725 | 16,32413 | 22.08.2015 |
| DJ 2024 (unpublished data) | *Phoxinellus dalmaticus* | Native | CROATIA | Adriatic Sea | Krka | Petrovo Polje (upprt part as Mirlović Polje) | Čikola spring (main) | 43,79725 | 16,32413 | 21.01.2018 |
| DJ 2024 (unpublished data) | *Phoxinellus dalmaticus* | Native | CROATIA | Adriatic Sea | Krka | Petrovo Polje (upprt part as Mirlović Polje) | Velika Kanjovača (one of smaller Čikola springs) | 43,79746 | 16,32469 | 15.10.2008 |
| DJ 2024 (unpublished data) | *Phoxinellus dalmaticus* | Native | CROATIA | Adriatic Sea | Krka | Petrovo Polje (upprt part as Mirlović Polje) | Mala Kanjovača (one of smaller Čikola springs) | 43,79701 | 16,32471 | 08.01.2015 |
| DJ 2024 (unpublished data) | *Phoxinellus dalmaticus* | Native | CROATIA | Adriatic Sea | Krka | Petrovo Polje lower part as Drniško Polje) | Middle and upper parts of Čikola in Drniško polje | 43,84992 | 16,12678 | before 2024 |
| DJ 2024 (unpublished data) | *Phoxinellus dalmaticus* | Native | CROATIA | Adriatic Sea | Krka |  | Cave opposite to Torak lake (located at estuary of Čikola to Krka) | 43,81817 | 16,01229 | before 2024 |
| NMW | *Phoxinellus pseudalepidotus* | Native | BiH | Adriatic Sea | Neretva | Mostarsko Blato | Mostrasko Blato: localities not specified | 43,31585 | 17,72892 | 25.02.1896 |
| PZ, Zupancic & Bogutskaya (2000) | *Phoxinellus pseudalepidotus* | Native | BiH | Adriatic Sea | Neretva | Mostarsko Blato | Lištica River at Troskoti | 43,32389 | 17,73666 | 2002; 2003; 2007 |
| **Source** | **ScientificName** | **Native Status** | **Country** | **Basin** | **Drainage** | **Polje** | **Locality** | **Latitude decimal** | **Longitude decimal** | **Date** |
| PZ, Zupancic & Bogutskaya (2000) | *Phoxinellus pseudalepidotus* | Native | BiH | Adriatic Sea | Neretva | Mostarsko Blato | Canal at Ljuti Dolac | 43,31365 | 17,71263 | 16.07.1998 |
| PZ, Zupancic & Bogutskaya (2000) | *Phoxinellus pseudalepidotus* | Native | BiH | Adriatic Sea | Neretva | Mostarsko Blato | at Ljuti Dolac | 43,31968 | 17,70437 | 22.05.1999, 22.05.2000 |
| PZ, Zupancic & Bogutskaya (2000) | *Phoxinellus pseudalepidotus* | Native | BiH | Adriatic Sea | Neretva | Mostarsko Blato | Canal Jare | 43,33111 | 17,65184 | 23.07.1997, 16.07.1998 |
| Markotić et al. (2019) | *Phoxinellus pseudalepidotus* | Native | BiH | Adriatic Sea | Neretva | Mostarsko Blato | Pisak | 43,32452 | 17,67318 | 2009 |
| Markotić et al. (2019) | *Phoxinellus pseudalepidotus* | Native | BiH | Adriatic Sea | Neretva | Mostarsko Blato | Međurić | 43,32038 | 17,66958 | 2009 |
| Markotić et al. (2019) | *Phoxinellus pseudalepidotus* | Native | BiH | Adriatic Sea | Neretva | Mostarsko Blato | Pološki Gaz | 43,33585 | 17,6825 | 2009 |
| DJ HDBI 1303 | *Phoxinellus pseudalepidotus* | Native | BiH | Adriatic Sea | Neretva | Mostarsko Blato | Mostrasko Blato: locality not specified |  |  | 2008 |
| DJ HDBI 1310 | *Phoxinellus pseudalepidotus* | Native | BiH | Adriatic Sea | Neretva | Mostarsko Blato | Mostrasko Blato: locality not specified |  |  | Sep 08 |

**Table S1b: *P. alepidotus***

| **Source of data** | **Scientific Name** | **Native Status** | **Country** | **Basin** | **Drainage** | **Polje** | **Locality** | **Latitude decimal** | **Longitude decimal** | **Date of field work** | **Comments:** |
| --- | --- | --- | --- | --- | --- | --- | --- | --- | --- | --- | --- |
| Geiger et al. (2014) | *Phoxinellus alepidotus* | Native | BiH | Adriatic Sea | Cetina | Grahovsko/Pašića Polje | Stream Korana (karstic) in Bosansko Grahovo | 44,17694 | 16,38417 |  |  |
| Maric (1981), PZ, Bogutskaya & Zupančič (2003) | *Phoxinellus alepidotus* | Native | BiH | Adriatic Sea | Cetina | Grahovsko/Pašića Polje | Stream Korana (karstic) in Bosansko Grahovo | 44,18293 | 16,35296 | before 1980; 14 May 2002, 11 May 2003 |  |
| Freyhof et al. (2006): FSJF 1025 | *Phoxinellus alepidotus* | Native | BiH | Adriatic Sea | Cetina | Grahovsko/Pašića Polje | Stream Korana, from Bosansko Grahovo south at bridge to village Obljaj | 44,16674 | 16,3855 | before 2006 |  |
| Marić (1980, 1983) | *Phoxinellus alepidotus* | Native | BiH | Black Sea | Danube | Grahovsko Polje | Source of Struga (Jaruga) in Bosansko Grahovo municipality (east of Resanovci and Ledina caves). According to Maric, belong to the Danube drainage. | 44,29601 | 16,3085 | before 1980 | Grahovsko polje is divided into the basins of the Black and Adriatic seas |
| DJ 2024 (ms) | *Phoxinellus alepidotus* | Native | BiH | ? | ? | Grahovsko Polje | spring Zvijezda at Bosansko Grahovo |  |  | 21 Sept 2008 | Grahovsko polje is divided into the basins of the Black and Adriatic seas |
| DJ 2024 (ms) | *Phoxinellus alepidotus* | **Introduced (appered from Satorsko Lake)** | BiH | Black Sea | Danube |  | Bulino vrelo springs of Unac River (tributary of Una) |  |  | 2 Oct 2008 | Grahovsko polje is divided into the basins of the Black and Adriatic seas |
| Delić et al. (2005) | *Phoxinellus alepidotus* | **Introduced from Bosansko Grahovo** | BiH | Black Sea | Danube |  | Šatorsko Lake | 44,16447 | 16,60172 | August 2003 and 2004 | introduced from Bosansko Grahovo, as published in Delić et al. 2005 |
| DJ_HDBI 261 | *Phoxinellus alepidotus* | **Introduced from Bosansko Grahovo** | BiH | Black Sea | Danube |  | Šatorsko Lake | 44,16447 | 16,60172 | 2013 | introduced from Bosansko Grahovo, as published in Delić et al. 2005 |
| DJ_HDBI 1219 | *Phoxinellus alepidotus* | **Introduced from Bosansko Grahovo** | BiH | Black Sea | Danube |  | Šatorsko Lake | 44,16447 | 16,60172 | 22.08.2010 | introduced from Bosansko Grahovo, as published in Delić et al. 2005 |
| Karaman (1972) | *Phoxinellus alepidotus* | Native | BiH | ? | ? | Glamočko Polje | Glamočko Polje (no exact locality) |  |  |  |  |
| Zupančič and Bogutskaya (2002) | *Phoxinellus alepidotus* | Native | BiH | Adriatic Sea | Cetina | Glamočko Polje | Donje Polje: Vrba River N of Glamoč, at Scucani | 43,98947 | 16,91723 | 12 Aug 2001 |  |
| **Source of data** | **Scientific Name** | **Native Status** | **Country** | **Basin** | **Drainage** | **Polje** | **Locality** | **Latitude decimal** | **Longitude decimal** | **Date of field work** | **Comments:** |
| Zupančič and Bogutskaya (2002) | *Phoxinellus alepidotus* | Native | BiH | ? | ? | Glamočko Polje | unnamed creek at Glamoč | 44,03232 | 16,8956 | 11 Jun 1999 |  |
| unpubl. data of P. Zupančič | *Phoxinellus alepidotus* | Native | BiH | ? | ? | Glamočko Polje | unnamed stream | 44,04234 | 16,90515 | 30 Jun 2004 |  |
| unpubl. data of P. Zupančič | *Phoxinellus alepidotus* | Native | BiH | ? | ? | Glamočko Polje | Stream Glamoč | 44,03232 | 16,8956 | 19 Aug 2009 |  |
| Zupančič and Bogutskaya (2002) | *Phoxinellus alepidotus* | Native | BiH | ? | ? | Glamočko Polje | stream 2 km SE of Glamoč | 44,03833 | 16,87861 | 11 Jun 1999 |  |
| Zupančič and Bogutskaya (2002) | *Phoxinellus alepidotus* | Native | BiH | Adriatic Sea | Cetina | Glamočko Polje | Donje Polje: Jaruga stream at eastern Glamoč | 44,04632 | 16,88572 | 12 Aug 1997 |  |
| Zupančič and Bogutskaya (2002) | *Phoxinellus alepidotus* | Native | BiH | Adriatic Sea | Cetina | Glamočko Polje | Donje Polje: Vrba stream | 43,98949 | 16,91754 | 12 Aug 1997 |  |
| Zupančič and Bogutskaya (2002) | *Phoxinellus alepidotus* | Native | CROATIA | Adriatic Sea | Cetina | Sinjsko Polje | Miloševo Jezero Lake, south of Hrvace (very south of Sinjsko polje) | 43,74524 | 16,63765 | 7 Aug 1999 | **DJ 2024: not confirmed in recent years** |
| Ćaleta et al. 2015 | *Phoxinellus alepidotus* | Native | CROATIA | Adriatic Sea | Cetina | Sinjsko Polje | Stipančevo Jezero Lake, south of Hrvace (very south of Sinjsko polje) | 43,74479 | 16,63906 | before 2015 (no date given) | **DJ 2024: not confirmed in recent years** |
| Ćaleta et al. 2015 | *Phoxinellus alepidotus* | Native | CROATIA | Adriatic Sea | Cetina | Sinjsko Polje | One location along Cetina River (on graphic map, near Sinj); coordinates are ca from map in Ćaleta et al. 2015 | 43,71868 | 16,70846 | before 2015 (no date given) | **no exact locality given** |
| NMW (several) | *Phoxinellus alepidotus* | Native | BiH | Adriatic Sea | Cetina | Sinjsko Polje | Sign (Sign region (?); coordinates not known |  |  | Historical samples | |
| Delić et al. (2005) with reference to Marić (1986) | *Phoxinellus alepidotus* | Native | BiH | Adriatic Sea | Cetina | Livanjsko polje | Ždralovac karst river and lake, very north-western part of Livanjsko polje | 44,08093 | 16,61579 | before 1986 |  |
| Perea et al. (2010), Schönhuth et al. (2018), NMP uncatalogued (B68) | *Phoxinellus alepidotus* | Native | BiH | Adriatic Sea | Cetina | Livanjsko polje | Ševarova Jaruga (NW of Livanjsko Polje: Ždralovac is drained by Ševarova Jaruga to its ponor near the village Donji Kazanci), now modified into Ždralovacki draining canal. | 44,0083 | 16,60682 | before 2018 (no date given) |  |
| NMW | *Phoxinellus alepidotus* | Native | BiH | Adriatic Sea | Cetina | Livanjsko polje | Livno (location not specified) |  |  | Historical samples | |
| Univ. Ljubljana uncat. | *Phoxinellus alepidotus* | Native | BiH | Adriatic Sea | Cetina | Livanjsko polje | Livno (location not specified) | 44,01140 | 16,61703 | August 1968 |  |
| **Source of data** | **Scientific Name** | **Native Status** | **Country** | **Basin** | **Drainage** | **Polje** | **Locality** | **Latitude decimal** | **Longitude decimal** | **Date of field work** | **Comments:** |
| DJ 2024 | *Phoxinellus alepidotus* | Native | BiH | Adriatic Sea | Cetina | Livanjsko polje | two small springs into Čoluša canal (Gornji Kazanci) |  |  | 12 Jun 2016 |  |
| Ćurčić (1916), Taler (1953), Sabioncello (1967), Vuković & Ivanović (1971),Vuković ( 1977,1982) | *Phoxinellus alepidotus* | Native | CROATIA | Adriatic Sea | Cetina | Duvanjsko Polje | **Duvanjsko Polje (main stream Šujica)** |  |  |  | **Neither exact literature data nor vouchers. Not found by PZ (1901-2007).** |
| Ćurčić (1916), Taler (1953), Sabioncello (1967), Vuković & Ivanović (1971),Vuković ( 1977,1982) | *Phoxinellus alepidotus* | Native | CROATIA | Adriatic Sea | Cetina | Duvanjsko Polje | **Blidinje Lake** |  |  |  | **Neither exact literature data nor vouchers. Not found by PZ (1901-2007).** |
| Zupančič (2008), Ćaleta et al. (2015) | *Phoxinellus alepidotus* | probably, originated from other sources in Livanjsko Polje | BiH | Adriatic Sea | Cetina | Livanjsko polje | **Buško Blato (Busko Lake), artificial, south-eastern part of Livanjsko Polje** | | |  |  |
| DJ 2024 |  |  | CROATIA |  | Cetina | Sinjsko Polje | **North side of Sinjsko polje incl. large springs, bringing water from Herzegovina (dived): Grab, Ruda, Vukovića vrelo, Miloševo vrelo, Rumin.** | | | Recent | **Not found by P. Zupančič (1991-2007; personal communication). Neither exact literature data nor vouchers.** |
| DJ 2024 |  |  | CROATIA |  | Cetina | Sinjsko Polje | **Small stream Sutina, and sorrounding water bodies, like spring Goručica (in Sinj)** | | | Recent | **Not found by P. Zupančič (1991-2007; personal communication). Neither exact literature data nor vouchers.** |

**2.2 Specimens used for DNA analysis**

**Table S2a: The complete mitochondrial genomes downloaded from GenBank.**

| **GenBank No.** | **Species** | **GenBank No.** | **Species** |
| --- | --- | --- | --- |
| AP018425 | *Abramis brama* | NC_029426 | *Leuciscus burdigalensis* |
| KC894466 | *Abramis brama* | NC_063524 | *Leuciscus idus* |
| MT410936 | *Abramis brama* | NC_069632 | *Leuciscus merzbacheri* |
| NC_020356 | *Abramis brama* | NC_029425 | *Leuciscus oxyrrhis* |
| ON123737 | *Abramis brama* | MN105127 | *Leuciscus waleckii* |
| OZ023101 | *Abramis brama* | NC_018825 | *Leuciscus waleckii* |
| NC_031562 | *Acanthobrama persidis* | NC_033920 | *Pachychilon pictum* |
| MT584105 | *Alburnus alburnus* | NC_008663 | *Pelecus cultratus* |
| NC_008659 | *Alburnus alburnus* | NC_031574 | *Pseudochondrostoma polylepis* |
| OM736796 | *Alburnus alburnus* | AP010775 | *Rutilus rutilus* |
| OM736798 | *Alburnus alburnus* | NC_068671 | *Rutilus rutilus* |
| KF534726 | *Alburnus chalcoides* | OM736800 | *Rutilus rutilus* |
| NC_019574 | *Alburnus istanbulensis* | OR263623 | *Rutilus rutilus* |
| NC_031573 | *Alburnus mossulensis* | OX637958 | *Rutilus rutilus* |
| NC_019575 | *Alburnus tarichi* | NC_031561 | *Scardinius erythrophthalmus* |
| NC_031563 | *Aspiolucius esocinus* | NC_031540 | *Squalius cephalus* |
| MT410960 | *Blicca bjoerkna* | OM736795 | *Squalius cephalus* |
| NC_020355 | *Blicca bjoerkna* | OM736799 | *Squalius cephalus* |
| NC_008108 | *Chondrostoma lemmingii* | OX439272 | *Squalius cephalus* |
| NC_084352 | *Iberochondrostoma almacai* | NC_008648 | *Tinca tinca* |
| NC_020357 | *Leucaspius delineatus* | NC_031539 | *Vimba melanops* |
| NC_024528 | *Leuciscus baicalensis* |  |  |

**Table S2b: The complete mitochondrial genomes downloaded from GenBank.**

| **LabID** | **Species** | **Type status** | **historical/**  **recent** | **fragment successfuly amplified** | **Source** | **Reference** | **NMW Number** | **Individum** | **Year** | **Locality** | **River Drainage** | **y** | **x** | **Country** | **Collected** |
| --- | --- | --- | --- | --- | --- | --- | --- | --- | --- | --- | --- | --- | --- | --- | --- |
| KJ554493 | P dalmaticus |  | recent* | COI complete | GenBank | Geiger et al. 2014 | / | Ex45E4 | 2014 | Krka drainage | Krka | 43.81719 | 16.26775 | Croatia |  |
| KJ554535 | P dalmaticus |  | recent* | COI complete | GenBank | Geiger et al. 2014 | / | Ex47H9 | 2014 | Krka drainage | Krka | 43.81719 | 16.26775 | Croatia |  |
| Pale89 | P alepidotus |  | historical | C2 | this study |  | 12971 | 1 | 1881 | Sinj*** | Cetina |  |  | Croatia | Steindachner |
| Pale59 | P alepidotus |  | historical | COI C1+C2 | this study |  | 51051 | 1 | 1881 | Sinj*** | Cetina |  |  | Croatia | Steindachner |
| Pale60 | P alepidotus |  | historical | COI C1+C2 | this study |  | 51051 | 2 | 1881 | Sinj*** | Cetina |  |  | Croatia | Steindachner |
| Pale61 | P alepidotus |  | historical | COI C1+C2 | this study |  | 51051 | 3 | 1881 | Sinj*** | Cetina |  |  | Croatia | Steindachner |
| Pale62 | P alepidotus |  | historical | COI C1+C2 | this study |  | 51051 | 4 | 1881 | Sinj*** | Cetina |  |  | Croatia | Steindachner |
| KJ554525 | P pseudoalepidotus | | recent | COI complete | GenBank | Geiger et al. 2014 | / | Ex01D7 | 2014 | Mostarsko Blato | Neretva (right bank) | 43.31365 | 17.71263 | Bosnia-Herzegovina | |
| HM560286 | P alepidotus |  | recent | COI complete | GenBank | Perea et al. 2010 | / | BOS352 | 2010 | Ševarova Jaruga | Cetina | 44.08093 | 16.61579 | Bosnia-Herzegovina | |
| HM560287 | P dalmaticus |  | recent* | COI complete | GenBank | Perea et al. 2010 | / | PH111 | 2010 | Krka drainage | Krka | 43.81719 | 16.26775 | Croatia |  |
| HM560288 | P pseudoalepidotus | | recent | COI complete | GenBank | Perea et al. 2010 | / | BOS107 | 2010 | Mostarsko Blato | Neretva (right bank) | 43.31365 | 17.71263 | Bosnia-Herzegovina | |
| MG806856 | P alepidotus |  | recent | COI complete | GenBank | Schonhuth et al. 2018 | / | B68 | 2018 | Ševarova Jaruga | Cetina | 44.08093 | 16.61579 | Bosnia-Herzegovina | |
| MG806857 | P pseudoalepidotus | | recent | COI complete | GenBank | Schonhuth et al. 2018 | / | B26 | 2018 | Lištica River, Mostarsko Blato | Neretva (right bank) | 43.32389 | 17.73666 | Bosnia-Herzegovina | |
| Pdal8 | P dalmaticus |  | historical | complete mtG | this study |  | 51053 | 1 | 1897 | Čikola River | Krka |  |  | Croatia | Kolombatovic |
| Tpol1 | Telestes polylepis | PARALECTOTYPE(S) | historical | complete mt genome, more data available | this study |  | 49710 | 1 | 1866 | Josipdol | Danube |  |  | Croatia | Postmeisters |
| Pale63 | P alepidotus |  | historical | COI C1+C2 | this study |  | 51051 | 5 | 1881 | Sinj*** | Cetina |  |  | Croatia | Steindachner |
| **LabID** | **Species** | **Type status** | **historical/**  **recent** | **fragment successfuly amplified** | **Source** | **Reference** | **NMW Number** | **Individum** | **Year** | **Locality** | **River Drainage** | **y** | **x** | **Country** | **Collected** |
| Pale55 | P alepidotus |  | historical | COI C1+C2 | this study |  | 51052 | 1 | 1881 | Sinj*** | Cetina |  |  | Croatia | Steindachner |
| Pale56 | P alepidotus |  | historical | COI C1+C2 | this study |  | 51052 | 2 | 1881 | Sinj*** | Cetina |  |  | Croatia | Steindachner |
| Pale57 | P alepidotus |  | historical | COI C1+C2 | this study |  | 51052 | 3 | 1881 | Sinj*** | Cetina |  |  | Croatia | Steindachner |
| Pale58 | P alepidotus |  | historical | COI C1+C2 | this study |  | 51052 | 4 | 1881 | Sinj*** | Cetina |  |  | Croatia | Steindachner |
| Pale10 | P alepidotus |  | historical | C1_F | this study |  | 51054 | 1 | 1883 | Sinj*** | Cetina |  |  | Croatia | Steindachner |
| Pale100 | P alepidotus |  | historical | COI C1+C2 | this study |  | 51054 | 1 | 1883 | Sinj*** | Cetina |  |  | Croatia | Steindachner |
| Pale101 | P alepidotus |  | historical | COI C1+C2 | this study |  | 51054 | 2 | 1883 | Sinj*** | Cetina |  |  | Croatia | Steindachner |
| Pale102 | P alepidotus |  | historical | COI C1+C2 | this study |  | 51054 | 3 | 1883 | Sinj*** | Cetina |  |  | Croatia | Steindachner |
| Pale103 | P alepidotus |  | historical | COI C1+C2 | this study |  | 51054 | 4 | 1883 | Sinj*** | Cetina |  |  | Croatia | Steindachner |
| Pale104 | P alepidotus |  | historical | COI C1+C2 | this study |  | 51054 | 5 | 1883 | Sinj*** | Cetina |  |  | Croatia | Steindachner |
| Pale105 | P alepidotus |  | historical | COI C1+C2 | this study |  | 51054 | 6 | 1883 | Sinj*** | Cetina |  |  | Croatia | Steindachner |
| Pale106 | P alepidotus |  | historical | COI C1+C2 | this study |  | 51054 | 7 | 1883 | Sinj*** | Cetina |  |  | Croatia | Steindachner |
| Pale107 | P alepidotus |  | historical | COI C1+C2 | this study |  | 51054 | 8 | 1883 | Sinj*** | Cetina |  |  | Croatia | Steindachner |
| Pale108 | P alepidotus |  | historical | COI C1+C2 | this study |  | 51054 | 9 | 1883 | Sinj*** | Cetina |  |  | Croatia | Steindachner |
| Ppse4 | P pseudoalepidotus | HOLOTYPE | historical | complete mtG | this study |  | 51102 | 1 | 1896 | Mostarsko Blato | Neretva (right bank) |  |  | Bosnia-Herzegovina | Hawelka |
| NEZ09-1 | Delminichthys adspersus | | historical | complete mt genome, more data available | this study |  | tissue only |  | 2010 | Nezdravica | Tihaljina |  |  | Bosnia-Herzegovina | |
| Pepir1 | Pelasgus epiroticus | SYNTYPES | historical | complete mt genome, more data available | this study |  | 51122 | 1 | 1892 | Pamvotis |  |  |  | Greece | Steindachner |
| **LabID** | **Species** | **Type status** | **historical/**  **recent** | **fragment successfuly amplified** | **Source** | **Reference** | **NMW Number** | **Individum** | **Year** | **Locality** | **River Drainage** | **y** | **x** | **Country** | |
| Pale17 | P alepidotus |  | historical | complete mtG | this study |  | 51057 | 1 | 1883 | Livno*** | Cetina |  |  | Bosnia-Herzegovina | |
| Pale18 | P alepidotus |  | historical | C1_F | this study |  | 51057 | 2 | 1883 | Livno*** | Cetina |  |  | Bosnia-Herzegovina | Unknown |
| Pale19 | P alepidotus |  | historical | COI C1+C2 | this study |  | 51057 | 3 | 1883 | Livno*** | Cetina |  |  | Bosnia-Herzegovina | Unknown |
| Pale20 | P alepidotus |  | historical | complete mtG | this study |  | 51057 | 4 | 1883 | Livno*** | Cetina |  |  | Bosnia-Herzegovina | Unknown |
| Pale21 | P alepidotus |  | historical | complete mtG | this study |  | 51057 | 5 | 1883 | Livno*** | Cetina |  |  | Bosnia-Herzegovina | Unknown |
| Pale22 | P alepidotus |  | historical | COI C1+C2 | this study |  | 51057 | 6 | 1883 | Livno*** | Cetina |  |  | Bosnia-Herzegovina | Unknown |
| Pale23 | P alepidotus |  | historical | C1_F | this study |  | 51057 | 7 | 1883 | Livno*** | Cetina |  |  | Bosnia-Herzegovina | Unknown |
| Pale24 | P alepidotus |  | historical | complete mtG | this study |  | 51057 | 8 | 1883 | Livno*** | Cetina |  |  | Bosnia-Herzegovina | Unknown |
| Pale25 | P alepidotus |  | historical | C1_F | this study |  | 51057 | 9 | 1883 | Livno*** | Cetina |  |  | Bosnia-Herzegovina | Unknown |
| Pale26 | P alepidotus |  | historical | COI C1+C2 | this study |  | 51057 | 10 | 1883 | Livno*** | Cetina |  |  | Bosnia-Herzegovina | Unknown |
| Pale27 | P alepidotus |  | historical | COI C1+C2 | this study |  | 51057 | 11 | 1883 | Livno*** | Cetina |  |  | Bosnia-Herzegovina | Unknown |
| Pale92 | P alepidotus |  | historical | C2 | this study |  | 51058 | 3 | 1883 | Livno*** | Cetina |  |  | Bosnia-Herzegovina | Unknown |
| Pale39 | P alepidotus |  | historical | C1_F | this study |  | 51059 | 3 | 1883 | Livno*** | Cetina |  |  | Croatia | Steindachner |
| Pale80 | P alepidotus |  | historical | COI C1+C2 | this study |  | 51062 | 1 | 1883 | Sinj*** | Cetina |  |  | Croatia | Steindachner |
| Pale81 | P alepidotus |  | historical | COI C1+C2 | this study |  | 51062 | 2 | 1883 | Sinj*** | Cetina |  |  | Croatia | Steindachner |
| **LabID** | **Species** | **Type status** | **historical/**  **recent** | **fragment successfuly amplified** | **Source** | **Reference** | **NMW Number** | **Individum** | **Year** | **Locality** | **River Drainage** | **y** | **x** | **Country** | **Collected** |
| Pale82 | P alepidotus |  | historical | COI C1+C2 | this study |  | 51062 | 3 | 1883 | Sinj*** | Cetina |  |  | Croatia | Steindachner |
| Pale83 | P alepidotus |  | historical | COI C1+C2 | this study |  | 51062 | 4 | 1883 | Sinj*** | Cetina |  |  | Croatia | Steindachner |
| Pale84 | P alepidotus |  | historical | COI C1+C2 | this study |  | 51062 | 5 | 1883 | Sinj*** | Cetina |  |  | Croatia | Steindachner |
| Pale85 | P alepidotus |  | historical | C1_F | this study |  | 51062 | 6 | 1883 | Sinj*** | Cetina |  |  | Croatia | Steindachner |
| Pale99 | P alepidotus |  | historical | COI C1+C2 | this study |  | 51107 | 3 | 1856 | Sinj*** | Cetina |  |  | Croatia | Bellotti |
| Pale64 | P alepidotus |  | historical | C1_F | this study |  | 51108 | 1 | 1881 | Sinj*** | Cetina |  |  | Croatia | Steindachner |
| Pale64 | P alepidotus |  | historical | complete mtG | this study |  | 51108 | 1 | 1881 | Sinj*** | Cetina |  |  | Croatia | Steindachner |
| Pale65 | P alepidotus |  | historical | COI C1+C2 | this study |  | 51108 | 2 | 1881 | Sinj*** | Cetina |  |  | Croatia | Steindachner |
| Pale66 | P alepidotus |  | historical | C1_F | this study |  | 51108 | 3 | 1881 | Sinj*** | Cetina |  |  | Croatia | Steindachner |
| Pale68 | P alepidotus |  | historical | C2 | this study |  | 51108 | 5 | 1881 | Sinj*** | Cetina |  |  | Croatia | Steindachner |
| Pdal2 | P dalmaticus |  | recent* | complete mtG | DNA used already in Reier et al. 2022 | | tissue only |  | 1990 | Čikola River | Krka | 43.81719 | 16.26775 | Croatia | Unknown |
| Pdal3 | P dalmaticus |  | recent* | complete mtG | DNA used already in Reier et al. 2022 | | tissue only |  | 1990 | Čikola River | Krka | 43.81719 | 16.26775 | Croatia | Unknown |
| Pdal5 | P dalmaticus |  | recent* | complete mtG | DNA used already in Reier et al. 2022 | | tissue only |  | 1990 | Čikola River | Krka | 43.81719 | 16.26775 | Croatia | Unknown |
| Pdal6 | P dalmaticus |  | recent* | complete mtG | DNA used already in Reier et al. 2022 | | tissue only |  | 1990 | Čikola River | Krka | 43.81719 | 16.26775 | Croatia | Unknown |
| Pale69 | P alepidotus |  | historical | C2 | this study |  | 51108 | 6 | 1881 | Sinj*** | Cetina |  |  | Croatia | Steindachner |
| Ppse1 | P pseudoalepidotus | | recent** | complete mtG | DNA used already in Reier et al. 2022 | | tissue only |  |  | Mostarsko Blato | Neretva (right bank) | 43.31365 | 17.71263 | Bosnia-Herzegovina | Prof. Ivan Bogut |
| Ppse2 | P pseudoalepidotus | | recent** | complete mtG | DNA used already in Reier et al. 2022 | | tissue only |  |  | Mostarsko Blato | Neretva (right bank) | 43.31365 | 17.71263 | Bosnia-Herzegovina | Prof. Ivan Bogut |
| Ppse3 | P pseudoalepidotus | | recent** | complete mtG | DNA used already in Reier et al. 2022 | | tissue only |  |  | Mostarsko Blato | Neretva (right bank) | 43.31365 | 17.71263 | Bosnia-Herzegovina | Prof. Ivan Bogut |
| Pale9 | P alepidotus |  | historical | complete mtG | this study |  | 51110 |  | 1883 | Sinj*** | Cetina |  |  | Croatia |  |
| **LabID** | **Species** | **Type status** | **historical/**  **recent** | **fragment successfuly amplified** | **Source** | **Reference** | **NMW Number** | **Individum** | **Year** | **Locality** | **River Drainage** | **y** | **x** | **Country** | **Collected** |
| Pale93 | P alepidotus |  | historical | COI C1+C2 | this study |  | 51111 | 1 | 1854 | Sinj*** | Cetina |  |  | Croatia | Frauenfeld |
| Pale96 | P alepidotus |  | historical | COI C1+C2 | this study |  | 51111 | 7 | 1854 | Sinj*** | Cetina |  |  | Croatia | Frauenfeld |

* wrong date given in the publication of Reier et al. 2022 - 1890 is acctually around 1990

** exact year unknown, but around 2000

*** exact localities unknown, only given as Sinj and Livno

**2.5 Specimens used for morphological analysis**

**TableS3:** Primary descriptive statistics for *Phoxinellus* material examined in this study. Abbreviations: CNHM, Croatian Natural History Museum, Zagreb; HDBI, Croatian Biology Research Society, Zagreb; MNCN, Museo Nacional de Ciencias Naturales, Madrid; NMW, Fish Collection of the Natural History Museum, Vienna.

|  | ***P. alepidotus***  **NMW, locality Sinj:** 51051-52 (9), 51054 (9), 51062 (6), 51107-11 (17), 51113 (2); **locality Livno**: 51057-59 (20); **syntypes (mixture of Livno and Sign)** 51061 (3), 51106 (2); **locality “Bosnia”** 51047 (3), 51055 (3). HDBI, locality **Šatorsko Lake**: 1219 (5) | | | | | ***P. dalmaticus***  CNHM 5387 (holotype and 5 paratypes); NMW 51053 (2) | | | | | ***P. pseudalepidotus***  NMW 51087 (20), 51100-102 (20) (holotype and paratype); HDBI 1303 (3) | | | | |
| --- | --- | --- | --- | --- | --- | --- | --- | --- | --- | --- | --- | --- | --- | --- | --- |
| **MEASUREMENTS** | n | min | max | **Mean** | SD | n | min | max | **Mean** | SD | n | min | max | **Mean** | SD |
| SL, mm | 70 | 29.2 | 98.5 | **60.9** | 11.53 | 8 | 46.8 | 59.2 | **53.1** | 4.65 | 43 | 55.3 | 101.8 | **83.6** | 13.23 |
| Body depth at dorsal-fin origin (% SL) | 70 | 19.1 | 28.5 | **23.1** | 1.71 | 8 | 22.3 | 26.5 | **23.7** | 1.36 | 43 | 19.1 | 24.2 | **22.0** | 1.10 |
| Depth of caudal peduncle (% SL) | 70 | 8.7 | 11.8 | **10.4** | 0.64 | 8 | 10.9 | 12.6 | **11.5** | 0.59 | 43 | 8.6 | 11.0 | **9.6** | 0.56 |
| Depth of caudal peduncle (% length of caudal peduncle) | 70 | 41.7 | 59.1 | **50.9** | 3.10 | 8 | 52.4 | 71.8 | **61.8** | 5.98 | 43 | 45.1 | 61.3 | **51.5** | 3.84 |
| Body width at dorsal-fin origin (% SL) | 70 | 9.7 | 14.7 | **12.0** | 0.97 | 8 | 13.1 | 15.8 | **14.3** | 0.97 | 43 | 9.3 | 13.4 | **11.5** | 1.07 |
| Caudal peduncle width (% SL) | 70 | 4.6 | 12.0 | **8.2** | 1.13 | 8 | 7.2 | 9.0 | **7.9** | 0.66 | 43 | 5.8 | 9.6 | **8.1** | 0.87 |
| Predorsal length (% SL) | 70 | 53.1 | 60.6 | **56.5** | 1.27 | 8 | 56.9 | 59.6 | **58.0** | 1.00 | 43 | 56.6 | 60.6 | **57.9** | 1.05 |
| Postdorsal length (% SL) | 70 | 32.6 | 37.8 | **35.7** | 1.13 | 8 | 31.6 | 35.7 | **33.3** | 1.30 | 43 | 30.5 | 35.4 | **33.5** | 1.05 |
| Prepelvic length (% SL) | 70 | 49.6 | 57.8 | **52.8** | 1.53 | 8 | 50.3 | 53.6 | **52.1** | 1.11 | 43 | 51.3 | 57.1 | **54.2** | 1.34 |
| Preanal length (% SL) | 70 | 68.3 | 76.6 | **72.2** | 1.78 | 8 | 68.5 | 71.5 | **70.0** | 1.02 | 43 | 70.5 | 76.7 | **73.5** | 1.57 |
| Pectoral – pelvic-fin origin length (% SL) | 70 | 23.7 | 35.0 | **27.2** | 1.89 | 8 | 25.1 | 27.9 | **26.7** | 0.91 | 43 | 25.7 | 29.6 | **27.6** | 1.06 |
| Pelvic – anal-fin origin length (% SL) | 70 | 17.9 | 24.6 | **20.3** | 1.27 | 8 | 17.5 | 19.6 | **18.7** | 0.67 | 43 | 18.1 | 21.6 | **19.9** | 0.87 |
| Pelvic – anal-fin origin length (% Pectoral – pelvic-fin origin length) | 70 | 56.1 | 90.3 | **74.7** | 6.67 | 8 | 64.1 | 75.1 | **70.2** | 4.10 | 43 | 64.5 | 81.5 | **72.0** | 4.59 |
| Caudal peduncle length (% SL) | 70 | 17.4 | 22.5 | **20.4** | 0.88 | 8 | 16.3 | 20.8 | **18.7** | 1.35 | 43 | 17.5 | 20.3 | **18.8** | 0.75 |
| Dorsal-fin base length (% SL) | 70 | 9.7 | 12.9 | **11.3** | 0.64 | 8 | 8.9 | 12.1 | **10.9** | 1.22 | 43 | 10.1 | 12.7 | **11.1** | 0.66 |
| Dorsal fin depth (% SL) | 70 | 14.6 | 22.5 | **19.5** | 1.71 | 8 | 18.2 | 21.6 | **19.9** | 1.20 | 43 | 15.8 | 21.9 | **18.3** | 1.09 |
|  | ***P. alepidotus***  **NMW, locality Sinj:** 51051-52 (9), 51054 (9), 51062 (6), 51107-11 (17), 51113 (2); **locality Livno**: 51057-59 (20); **syntypes (mixture of Livno and Sign)** 51061 (3), 51106 (2); **locality “Bosnia”** 51047 (3), 51055 (3). HDBI, locality **Šatorsko Lake**: 1219 (5) | | | | | ***P. dalmaticus***  CNHM 5387 (holotype and 5 paratypes); NMW 51053 (2) | | | | | ***P. pseudalepidotus***  NMW 51087 (20), 51100-102 (20) (holotype and paratype); HDBI 1303 (3) | | | | |
| Anal-fin base length (% SL) | 70 | 8.3 | 11.3 | **9.8** | 0.68 | 8 | 8.8 | 13.4 | **10.7** | 1.44 | 43 | 8.5 | 11.6 | **9.9** | 0.63 |
| Anal fin depth (% SL) | 70 | 10.1 | 16.7 | **14.3** | 1.28 | 8 | 12.6 | 17.4 | **15.1** | 1.77 | 43 | 12.1 | 18.5 | **13.9** | 1.14 |
| Pectoral fin length (% SL) | 69 | 14.1 | 20.5 | **17.5** | 1.29 | 8 | 15.3 | 18.4 | **16.6** | 1.09 | 43 | 14.7 | 20.4 | **17.4** | 1.07 |
| Pectoral fin length (% Pectoral – pelvic-fin origin length) | 69 | 48.9 | 78.7 | **64.5** | 6.99 | 8 | 56.1 | 66.3 | **62.1** | 4.01 | 43 | 52.3 | 73.7 | **62.9** | 4.95 |
| Pelvic fin length (% SL) | 70 | 11.4 | 16.7 | **14.6** | 1.15 | 8 | 12.8 | 15.0 | **13.8** | 0.77 | 43 | 12.0 | 15.3 | **13.7** | 0.86 |
| Pelvic fin length (% Pelvic – anal-fin origin length) | 70 | 56.0 | 87.9 | **72.2** | 7.45 | 8 | 68.6 | 82.5 | **73.8** | 4.61 | 43 | 61.2 | 81.8 | **69.1** | 4.98 |
| Head length (% SL) | 70 | 24.7 | 30.3 | **26.6** | 1.09 | 8 | 27.9 | 30.1 | **28.9** | 0.89 | 43 | 25.6 | 29.2 | **26.8** | 0.84 |
| Head length (% body depth) | 70 | 94.0 | 137.3 | **115.7** | 8.61 | 8 | 107.8 | 133.6 | **122.2** | 7.94 | 43 | 110.4 | 144.1 | **121.9** | 6.70 |
| Head depth at nape (% SL) | 70 | 15.7 | 19.0 | **17.4** | 0.67 | 8 | 17.4 | 19.3 | **18.2** | 0.74 | 43 | 15.9 | 18.9 | **17.4** | 0.63 |
| Head depth at nape (% HL) | 70 | 60.0 | 71.0 | **65.5** | 2.32 | 8 | 59.8 | 66.8 | **63.2** | 2.44 | 43 | 60.9 | 68.5 | **64.8** | 1.83 |
| Head depth through eye (% HL) | 70 | 45.2 | 58.7 | **50.0** | 2.35 | 8 | 44.5 | 51.0 | **47.7** | 2.61 | 43 | 44.2 | 52.5 | **47.6** | 1.81 |
| Maximum head width (% SL) | 70 | 11.3 | 14.8 | **12.4** | 0.71 | 8 | 13.0 | 15.7 | **14.2** | 0.88 | 43 | 11.8 | 13.8 | **12.8** | 0.44 |
| Maximum head width (% HL) | 70 | 42.6 | 53.0 | **46.6** | 2.00 | 8 | 45.5 | 52.7 | **49.2** | 2.67 | 43 | 45.0 | 50.4 | **47.7** | 1.26 |
| Snout length (% SL) | 70 | 6.8 | 9.0 | **7.7** | 0.43 | 8 | 7.7 | 8.7 | **8.1** | 0.39 | 43 | 6.8 | 8.7 | **7.8** | 0.45 |
| Snout length (% HL) | 70 | 26.5 | 32.8 | **28.9** | 1.40 | 8 | 27.1 | 29.3 | **28.0** | 0.93 | 43 | 25.9 | 31.9 | **29.0** | 1.54 |
| Eye horizontal diameter (% SL) | 70 | 4.7 | 7.2 | **6.2** | 0.44 | 8 | 5.7 | 6.8 | **6.1** | 0.37 | 43 | 4.6 | 7.3 | **5.8** | 0.58 |
| Eye horizontal diameter (% HL) | 70 | 18.7 | 26.6 | **23.3** | 1.34 | 8 | 19.9 | 23.4 | **21.2** | 1.30 | 43 | 17.7 | 26.3 | **21.6** | 1.91 |
| Eye horizontal diameter (% interorbital width) | 70 | 59.2 | 90.0 | **74.0** | 5.85 | 8 | 60.4 | 78.2 | **66.0** | 6.62 | 43 | 56.3 | 88.1 | **70.5** | 6.77 |
| Postorbital distance (% HL) | 70 | 48.2 | 57.5 | **53.3** | 1.95 | 8 | 48.9 | 54.7 | **52.3** | 2.03 | 43 | 45.8 | 55.2 | **51.9** | 1.98 |
|  | ***P. alepidotus***  **NMW, locality Sinj:** 51051-52 (9), 51054 (9), 51062 (6), 51107-11 (17), 51113 (2); **locality Livno**: 51057-59 (20); **syntypes (mixture of Livno and Sign)** 51061 (3), 51106 (2); **locality “Bosnia”** 51047 (3), 51055 (3). HDBI, locality **Šatorsko Lake**: 1219 (5) | | | | | ***P. dalmaticus***  CNHM 5387 (holotype and 5 paratypes); NMW 51053 (2) | | | | | ***P. pseudalepidotus***  NMW 51087 (20), 51100-102 (20) (holotype and paratype); HDBI 1303 (3) | | | | |
| Interorbital width (% SL) | 70 | 7.2 | 9.6 | **8.4** | 0.43 | 8 | 8.1 | 10.3 | **9.3** | 0.77 | 43 | 7.5 | 9.1 | **8.2** | 0.42 |
| Interorbital width (% HL) | 70 | 27.8 | 35.8 | **31.5** | 1.51 | 8 | 28.7 | 34.9 | **32.2** | 2.20 | 43 | 28.4 | 33.1 | **30.7** | 1.20 |
| Length of upper jaw (% HL) | 70 | 23.9 | 32.1 | **27.9** | 1.29 | 8 | 21.3 | 23.6 | **22.7** | 0.88 | 43 | 25.1 | 30.8 | **27.9** | 1.40 |
| Length of upper jaw (% SL) | 70 | 6.3 | 8.2 | **7.4** | 0.30 | 8 | 6.1 | 6.9 | **6.6** | 0.27 | 43 | 6.7 | 8.2 | **7.5** | 0.40 |
| Length of lower jaw (% SL) | 70 | 7.4 | 11.2 | **9.7** | 0.74 | 8 | 8.8 | 10.4 | **9.6** | 0.59 | 43 | 8.8 | 10.4 | **9.7** | 0.36 |
| Length of lower jaw (% HL) | 70 | 28.2 | 40.5 | **36.4** | 2.12 | 8 | 31.4 | 34.9 | **33.1** | 1.49 | 43 | 33.6 | 39.0 | **36.3** | 1.02 |
| Length of lower jaw (% interorbital width) | 70 | 87.4 | 142.8 | **115.8** | 9.38 | 8 | 90.0 | 113.8 | **103.0** | 7.33 | 43 | 108.9 | 127.7 | **118.5** | 5.25 |
| Length of lower jaw (% depth of operculum) | 70 | 79.6 | 104.8 | **96.4** | 3.01 | 8 | 97.9 | 109.1 | **102.3** | 3.67 | 43 | 92.3 | 108.9 | **100.9** | 3.99 |
| Depth of operculum (% HL) | 70 | 34.0 | 43.1 | **37.7** | 2.07 | 8 | 31.0 | 34.7 | **32.4** | 1.54 | 43 | 33.8 | 39.2 | **36.0** | 1.37 |
| Interorbital width/eye horizontal diameter | 70 | 1.1 | 1.7 | **1.4** | 0.11 | 8 | 1.3 | 1.7 | **1.5** | 0.14 | 43 | 1.1 | 1.8 | **1.4** | 0.14 |
| Snout length/eye horizontal diameter | 70 | 1.1 | 1.6 | **1.2** | 0.08 | 8 | 1.2 | 1.5 | **1.3** | 0.07 | 43 | 1.0 | 1.8 | **1.4** | 0.15 |
| Head depth /eye horizontal diameter | 70 | 2.4 | 3.5 | **2.8** | 0.18 | 8 | 2.7 | 3.2 | **3.0** | 0.18 | 43 | 2.5 | 3.7 | **3.0** | 0.26 |
| Head length/caudal peduncle depth | 70 | 2.2 | 3.4 | **2.6** | 0.20 | 8 | 2.3 | 2.6 | **2.5** | 0.10 | 43 | 2.4 | 3.2 | **2.8** | 0.18 |
| Length of caudal peduncle/caudal peduncle depth | 70 | 1.7 | 2.4 | **2.0** | 0.13 | 8 | 1.4 | 1.9 | **1.6** | 0.16 | 43 | 1.6 | 2.2 | **2.0** | 0.14 |
| Length of lower jaw/caudal peduncle depth | 70 | 0.7 | 1.2 | **0.9** | 0.09 | 8 | 0.8 | 0.9 | **0.8** | 0.04 | 43 | 0.8 | 1.1 | **1.0** | 0.07 |
| Pectoral fin length/pectoral – pelvic-fin origin distance | 69 | 0.5 | 0.8 | **0.6** | 0.07 | 8 | 0.6 | 0.7 | **0.6** | 0.04 | 43 | 0.5 | 0.7 | **0.6** | 0.05 |
| Predorsal length/head length | 70 | 1.9 | 2.3 | **2.1** | 0.09 | 8 | 1.9 | 2.1 | **2.0** | 0.06 | 43 | 2.0 | 2.3 | **2.2** | 0.06 |

|  | ***P. alepidotus***  **NMW, locality Sinj:** 12970-71 (2), 51051-52 (9), 51054 (9), 51062 (6), 51107-11 (17), 51113 (2); **locality Livno**: 51048-50 (11), 51056-60 (28); **syntypes (mixture of Livno and Sign) and localities not clearly specified** 51061 (3), 51106 (2); 51047 (3), 51055 (3). HDBI, locality **Šatorsko Lake**: 261 (2), 1219 (7) | | | | | ***P. dalmaticus***  CNHM 5387 (holotype and 5 paratypes); NMW 51053 (2); MNCN uncat. (collection of Primoz Zupancic, 18) | | | | | ***P. pseudalepidotus***  NMW 51087 (20), 51096-105 (118); HDBI 1303 (3) | | | | |
| --- | --- | --- | --- | --- | --- | --- | --- | --- | --- | --- | --- | --- | --- | --- | --- |
| **COUNTS** | n | min | max | **Mean** | SD | n | min | max | **Mean** | SD | n | min | max | **Mean** | SD |
| SL, mm | 104 | 29.2 | 98.5 | **69.8** | 12.33 | 26 | 42.0 | 59.2 | **52.1** | 4.45 | 141 | 40.7 | 102.7 | **84.5** | 13,37 |
| Number of branched dorsal-fin rays (without 1/2) | 104 | 7 | 8 | **7.0** | 0.11 | 26 | 7 | 7 | **7.0** | 0.00 | 141 | 7 | 8 | **7.0** | 0.15 |
| Number of branched anal-fin rays (without 1/2) | 104 | 7 | 8 | **7.1** | 0.31 | 26 | 7 | 7 | **7.0** | 0.00 | 141 | 6 | 8 | **7.0** | 0.24 |
| Number of predorsal vertebrae | 104 | 13 | 16 | **14.4** | 0.62 | 26 | 12 | 14 | **13.1** | 0.47 | 141 | 13 | 15 | **14.2** | 0.50 |
| Number of abdominal vertebrae | 104 | 21 | 23 | **22.4** | 0.59 | 26 | 20 | 22 | **20.9** | 0.42 | 141 | 21 | 23 | **22.4** | 0.56 |
| Number of caudal vertebrae | 104 | 15 | 19 | **16.7** | 0.75 | 26 | 16 | 17 | **16.3** | 0.46 | 141 | 16 | 18 | **16.5** | 0.52 |
| Total vertebrae | 104 | 37 | 41 | **39.2** | 0.78 | 26 | 36 | 38 | **37.3** | 0.51 | 141 | 38 | 40 | **38.9** | 0.63 |
| Total lateral-line scales | 104 | 10 | 60 | **29.8** | 10.08 | 26 | 16 | 29 | **22.5** | 4.11 | 141 | 19 | 73 | **57.8** | 9.94 |
| Total gill rakers (on outer side of first left gill arch) | 104 | 8 | 11 | **9.3** | 0.66 | 26 | 8 | 10 | **9.2** | 0.51 | 141 | 8 | 14 | **9.1** | 0.86 |

**3. Results**

**3.1 Notes on *Phoxinellus* ranges, sympatric (fish) species, subterranean and conservation status** (summarized in Table 2)

*P. dalmaticus*

Historically (NMW 51053) and in recent years (Zupančič & Bogutskaya 2000, Ćaleta et al. 2015, 2019), *P. dalmaticus* is restricted to the upper reaches of the Čikola River and its tributary Vrba River flowing through Petrovo Polje toward/into the Krka River (Adriatic Sea drainage basin). However, it has never been recorded in area of estuary where Čikola River discharges into Krka River, or in Krka itself.

Besides *P. dalmaticus*, *Telestes tursky* and *Aulopyge huegelii*, are native species of this area. From introduced species, *Lepomis gibbosus* was observed (personal observations NB, DJ).

*P. dalmaticus* has been observed in high numbers in the main Čikola spring during cave dives and population monitoring (DJ, 22.08.2015., 12 °C; 21.01.2018., 11 °C). This cave system is 300 m long, with the channel averaging 4 m high and 6-8 m wide before reaching the horizontal restriction, where the cave height averages 0.5-1 m high, but is very wide (>10 m). Channel is strongly flushed, with only large rocks and pebbles present (almost completely without sediment). The maximum depth at the restriction was 28.2 m (at high water). Individuals were also observed emerging from two smaller springs of Čikola (Velika Kanjovača, Mala Kanjovača; DJ, 15.10.2008; DJ 08.01.2015). Čikola River dries up almost completely in summer (July, August) and the population is maintained mainly by retreating underground. Part of the population remains stranded in depressions in the riverbed called "graba". These depressions are formed by the water flow in the soft alluvial deposits in the middle and upper part of Čikola. In the canyon, they are formed in the bedrock by whirlpools of water and rocks.

In 2004, this species was assessed by International Union for Conservation of Nature (IUCN) as Critically Endangered (CR) under criteria B1ab(ii)+2ab(ii) (Crivelli 2006b). A re-assessment has been recently applied to IUCN (Jelić & Freyhof 2024b, in submission) as follows: *P. dalmaticus* has a restricted range (extent of occurrence (EOO) ca 615 km2; Fig. 2A), which meets the threshold for the Endangered category under Criterion B1 (EOO < 5,000 km2). It is present at two locations where the extent and quality of habitat are estimated to be declining. Therefore, this species is re-assessed as Endangered (EN) under Criterion B (B1ab(iii)).

*P. alepidotus*

In Grahovo Polje, where the species was numerous over 50 years ago (Marić 1980, 1983), specimens of *P. alepidotus* were last observed in 2008 (DJ; see also Table 2). In addition, Delić et al. (2005) reported the collection of more than 40 specimens in Šatorsko Lake in August 2003 and 2004, which, according to the same reference, are a consequence of unintentional introduction in the 1970s-1980s; *P. alepidotus* was last observed in this lake in 2013 by DJ. In addition, it was also observed by DJ in the upper most areas of Unac River (Black Sea drainage basin; see also below).

The last published record of *P. alepidotus* in Glamočko Polje was in 2001 (Zupančič and Bogutskaya 2002), and the latest findings are from 2004 and 2009 (P. Zupančič, personal communication); these specimens were possibly collected in the central part of the polje, which drains into the Pliva-Vrbas-Danube drainage (Black Sea drainage basin) (also see Table S1).

Duvanjsko Polje and Blidinje Lake were included in the range of *P. alepidotus* by some authors. However, Zupančič and Bogutskaya (2002), based on field observations and intensive sampling by Primoz Zupančič, suggest that *P. alepidotus* is probably absent from Duvanjsko Polje and Blidinje Lake.

Historical specimens in the NHMW Fish Collection do not have exact localities, except for "Sign" (at present, in Croatian spelling Sinj) and "Livno", and no additional clues were found in the search of historical records in the collection.

In Sinjsko Polje, *P. alepidotus* was last confirmed in Stipančevo and Miloševo lakes by P. Zupančič during his field studies (Zupančič and Bogutskaya 2002), but has not been confirmed in recent years (DJ).

Locations on the northern side of Sinjsko Polje were investigated, and large springs that bring water from Herzegovina (Bosnia-Herzegovina) were dived (i.e. Grab, Ruda, Vukovića vrelo, Miloševo vrelo, Rumin), but no records of *Phoxinellus* were made. In addition to the two lakes mentioned above, *P. alepidotus* was also searched for in other small springs and streams in the south of Sinjsko Polje (e.g., stream Sutina, spring Goručica), but none were recorded.

During studies in 2002 and 2003, Primoz Zupancic did not collect *P. alepidotus* in any locality of Livanjsko Polje, but Delić et al. (2005), referring to Marić (1986) (not seen by us), reported *P. alepidotus* in two Ždralovac lakes (Mali and Veliki Ždralovac) in this polje. The most recent published records of *P. alepidotus* in NW of Livanjsko Polje (with voucher specimens) (Perea et al. 2010, Schönhuth et al. 2018) are from the karst stream Ševarova Jaruga which drained water of Ždralovac to its ponor near village of Donji Kazanci.

In Miloševo Lake in Sinjsko Polje, *P. alepidotus* was found together with the native *Aulopyge huegelii* and the introduced *Cyprinus carpio* and *Pseudorasbora parva* (Zupančič and Bogutskaya 2002). Besides *P. alepidotus*, *Telestes ukliva* is native to Sinjsko Polje, but it was never found in the same water bodies (Stipančevo and Miloševo lakes), nor in other poljes of *P. alepidotus* species range (Livanjsko, Grahovo, Glamočko). The only stygobiont vertebrate of the area, the cave salamander (*Proteus anguinus*), has not been recorded in any of the known *P. alepidotus* sites.

*P. alepidotus* has been recorded to enter the Zvijezda spring at Bosansko Grahovo (DJ, 21.09.2008) in Grahovo Polje, and the Bulino vrelo springs of the Unac River (tributary of the Una, Danube, Black Sea drainage basin; DJ, 02.10.2008), where it arrived through an underground connection from Šatorsko Lake. It has also been recorded to come out of two small springs into the Čoluša channel in Livanjsko Polje (DJ, 12.06.2016).

In 2004, this species was assessed by IUCN as Endangered (EN) under criteria B2ab(ii,iii,iv) (Crivelli 2006a). A re-assessment has been recently applied to IUCN (Jelić & Freyhof 2024a, in submission) as follows: *P. alepidotus* has a restricted range (EOO ca 2,643 km2), which meets the threshold for the Endangered category under Criterion B1 (EOO < 5,000 km2). It occurs in four locations where the extent and quality of habitat are estimated to be declining. Therefore, this species is assessed as Endangered under criteria B1ab(iii)) at the global and European regional scales. In the EU 27 member states, the EOO is ca 90 km2, which meets the threshold for the Critically Endangered category (EOO < 100 km2) and it is present at one location where the quality of habitat is estimated to be declining. It is therefore assessed as Critically Endangered under criteria B1ab(iii)). No downlisting of this category is deemed appropriate, since there is no possibility of genetic exchange with the remainder of the global population***.***

*P. pseudalepidotus*

From historical material held at NHMW and later on (Vuković and Ivanović 1971, Vuković 1977 (at that time still under the name *P. alepidotus*), Zupančič 2008), *P. pseudalepidotus* was always restricted to Mostarsko Blato with the Lištica River (Neretva drainage, Adriatic Sea drainage basin). DJ and DM visited Mostarsko Blato in 2008, and at that time *P. pseudalepidotus* was present in large numbers at eight different springs and small streams along the Lištica River. *P. pseudalepidotus* was 95% of the fish caught, the remaining 5% were native *Cobitis hercegoviensis* and introduced *Gambusia holbrooki*, *Oncorhynchus mykiss* and *Salmo trutta.* In 2017, the sites were revisited and *P. pseudalepidotus* was significantly reduced and even rare at some of the sites. In 2020, DM confirmed that the species is rare in the whole Lištica River, possibly due to high oscillations of water level caused by the the Mostarsko Blato hydroelectric power plant. As observed, *P.* *pseudalepidotus* spawns in shallow waters, which due to water regulations by the power plant may dry up and cause fish roes to dry as well. DM also reports the problem of illegal fishing, although protected by law, *P. pseudalepidotus* is still considered a delicacy by the local community.

Besides *P. pseudalepidotus*, the native fish fauna of Mostarsko Blato also includes Salmo obtusirostris, Anguilla anguilla (but both reported absent for 50 years), Salmo cf. farioides, and Cobitis hercegoviensis. There are also a number of introduced species, such as Salvelinus fontinalis, Oncorhynchus mykiss, Ameiurus nebulosus, Cyprinus carpio and Lepomis gibbosus which have become more common over the years (as observed during three different visits to the site between 2008 and 2020). The latter, L. gibbosus, seems to be the most intrusive and is gradually displacing *P. pseudalepidotus* from its natural habitat. There are no records of *Proteus anguinus* for Mostarsko Blato, but there are records for the nearby sites (e.g., Jasenica spring which brings water from Mostarsko Blato to the Neretva River), where no *P. pseudalepidotus* was recorded.

There is no evidence that *P. pseudalepidotus* occurs underground. DJ dived in the Bilo Vrilo cave in the town of Dobrič (SE of Široki Brijeg), which is one of the sources of Žvatić rivulet, but no fish were observed. There are no information for cave systems in the upper Lištica tributaries where the species could be expected to occur. It is important to mention that, in recent years, it appeared in small artificial ponds, created by digging clay, not connected to any watercourse.

In 2004, *P. pseudalepidotus* was assessed by IUCN as Vulnerable (VU) under criteria D2 (Crivelli 2006a). A re-assessment has been recently applied to IUCN (Jelić & Freyhof 2024c, in submission) as follows: *P. pseudalepidotus* has a restricted range (EOO ca 419 km2, area of occupancy (AOO) ca 40 km2; Fig. 2C), which meets the thresholds for the Endangered category under criteria B1 (EOO < 5,000 km2) and B2 (AOO < 500 km2). It is present at one location where the extent and quality of habitat are estimated to be declining. Therefore, this species is assessed as Endangered under criteria B (B1ab(iii)+2ab(iii)). EU 27 regional assessment: not recorded.

**3.4 Analysis of the nuclear DNA**

**Table S4:** Summary of main genome assembly metrics of *P. pseudalepidotus* revealed by BBmap v38.90 Bushnell (2014)

| **Metric** | **Value** |
| --- | --- |
| Main genome scaffold total: | 205092 |
| Main genome contig total: | 205092 |
| Main genome scaffold sequence total: | 783.209 MB |
| Main genome contig sequence total: | 783.209 MB 0.000% gap |
| Main genome scaffold N/L50: | 28264/8.036 KB |
| Main genome contig N/L50: | 28264/8.036 KB |
| Main genome scaffold N/L90: | 101106/2.11 KB |
| Main genome contig N/L90: | 101106/2.11 KB |
| Max scaffold length: | 85.355 KB |
| Max contig length: | 85.355 KB |
| Number of scaffolds > 50 KB: | 46 |
| % main genome in scaffolds > 50 KB: | 0.35% |

**Table S5:** BUSCO analysis of main genome assembly of *P. pseudalepidotus*. The analysis was conducted on a total of 3,640 BUSCO groups of the Actinopterygii dataset.

| **Metric** | **Value** |
| --- | --- |
| Complete BUSCOs (C) | 47.2% |
| Single-copy BUSCOs (S) | 46.3% |
| Duplicated BUSCOs (D) | 0.9% |
| Fragmented BUSCOs (F) | 13.8% |
| Missing BUSCOs (M) | 39.0% |
| Total BUSCO groups searched (n) | 3,640 |
| Number of Complete BUSCOs | 1,720 |
| Number of Complete and single-copy BUSCOs | 1,686 |
| Number of Complete and duplicated BUSCOs | 34 |
| Number of Fragmented BUSCOs | 502 |
| Number of Missing BUSCOs | 1,418 |

**Table S6**: Factor loadings DFA (Correlations Variables – Canonical Roots) (Fig. 8 in the main text) for three *Phoxinellus* species.

| Variable | Root 1 | Root 2 |
| --- | --- | --- |
| Lateral-line scales (total) | -0.023 | -0.655 |
| Caudal peduncle length | 0.257 | 0.156 |
| Upper jaw length | 0.215 | -0.086 |
| Head length (HL) | -0.221 | -0.108 |
| Caudal peduncle depth | -0.095 | 0.175 |
| Postdorsal length | 0.258 | 0.178 |
| Total vertebrae | 0.227 | -0.040 |
| Eye horizontal diameter | 0.082 | -0.133 |
| Postorbital distance | -0.108 | 0.104 |
| Predorsal length | -0.160 | -0.138 |
| Operculum depth | 0.164 | -0.111 |
| Pectoral fin length | 0.081 | -0.145 |
| Body depth maximal (in front of pelvic-fin origin) | 0.002 | 0.048 |
| Preanal length | 0.086 | -0.099 |
| Pelvic fin length | 0.124 | -0.064 |
| Number of branched anal-fin rays (without 1/2) | 0.046 | 0.040 |
| Lower jaw length | 0.026 | -0.189 |
| Anal-fin base length | -0.069 | -0.009 |
| Anal-fin depth | -0.002 | -0.130 |
| Number of abdominal vertebrae | 0.195 | -0.151 |
| Pectoral – pelvic-fin origin distance | 0.017 | 0.042 |
| Number of branched dorsal-fin rays (without 1/2) | 0.007 | -0.023 |
| Head depth at nape | -0.106 | -0.082 |
| Pelvic – anal-fin origin distance | 0.138 | 0.026 |
| Maximum head width | -0.266 | -0.064 |
| Prepelvic length | 0.000 | -0.070 |
| Gill rakers | 0.030 | -0.014 |
| Caudal peduncle width | 0.027 | 0.035 |
| Number of predorsal vertebrae | 0.185 | -0.099 |
| Body width at dorsal-fin origin | -0.165 | 0.133 |
| Head depth through eye | -0.007 | 0.008 |
| Dorsal-fin depth | 0.013 | -0.050 |
| Snout length | -0.088 | -0.057 |
| Interorbital width | -0.161 | 0.040 |
| Dorsal-fin base length | 0.060 | 0.016 |

**Table S7:** Factor loadings (unrotated) PCA (Fig. 9A in the main text) for four groups of *P. alepidotus* specimens.

| Variable | Factor 1 | Factor 2 |
| --- | --- | --- |
| Snout length | -0.589 | 0.269 |
| Eye horizontal diameter | -0.311 | 0.159 |
| Postorbital distance | -0.446 | 0.551 |
| Head length (HL) | -0.575 | 0.637 |
| Head depth at nape | -0.555 | 0.151 |
| Head depth through eye | -0.506 | 0.107 |
| Maximum head width | -0.567 | 0.353 |
| Interorbital width | -0.384 | 0.254 |
| Upper jaw length | -0.357 | 0.449 |
| Lower jaw length | -0.369 | 0.625 |
| Operculum depth | -0.281 | 0.601 |
| Body depth maximal (in front of pelvic-fin origin) | -0.191 | -0.180 |
| Caudal peduncle depth | -0.218 | -0.549 |
| Body width at dorsal-fin origin | -0.573 | -0.501 |
| Caudal peduncle width | -0.621 | -0.511 |
| Predorsal length | -0.148 | 0.461 |
| Postdorsal length | -0.194 | -0.027 |
| Prepelvic length | 0.011 | 0.467 |
| Preanal length | -0.133 | 0.302 |
| Caudal peduncle length | 0.087 | -0.343 |
| Dorsal-fin base length | -0.461 | -0.292 |
| Dorsal-fin depth | -0.749 | -0.344 |
| Anal-fin base length | -0.452 | -0.561 |
| Anal-fin depth | -0.561 | -0.461 |
| Pectoral fin length | -0.554 | -0.303 |
| Pelvic fin length | -0.557 | -0.398 |
| Pectoral – pelvic-fin origin distance | 0.230 | 0.062 |
| Pelvic – anal-fin origin distance | 0.014 | 0.151 |
| Number of branched dorsal-fin rays (without 1/2) | -0.177 | 0.184 |
| Number of branched anal-fin rays (without 1/2) | -0.038 | 0.451 |
| Number of predorsal vertebrae | -0.055 | 0.146 |
| Number of abdominal vertebrae | 0.100 | 0.042 |
| Number of caudal vertebrae | 0.220 | -0.167 |
| Total vertebrae | 0.301 | -0.138 |
| Lateral-line scales (total) | 0.037 | 0.008 |
| Gill rakers | 0.118 | 0.063 |

**References**

Andabaka, D., Senta Marić A., Gudelj, I. (2021). Čikola. In: Raspor, B (Ed.). Zagreb: Hrvatske vode, Institut Ruđer Bošković. P. 101–112.

Baučić, I. (1967). Cetina – razvoj reljefa i cirkulacija vode u kršu (Cetina – development of relief and water circulation in karst). Acta Geographica Croatica, 6(1), 5–167.

Bogutskaya, N. G. and P. Zupančič (2003) *Phoxinellus pseudalepidotus* (Telostei: Cyprinidae), a new species from the Neretva basin with an overview of the morphology of *Phoxinellus* species of Croatia and Bosnia-Herzegovina. Ichthyological Exploration of Freshwaters v. 14 (no. 4): 359–383.

Bonacci O., Ljubenkov I. (2005) New insights into the Krka river hydrology. Hrvatske Vode, 13 (52), 265–281.

Bonacci, O., Terzić, J., Roje-Bonacci, T. (2018). Hydrologka analiza krske rijeke Cikole. Hrvatske Vode, 26(106), 281–292.

Bushnell, B. (2014). BBMap: A fast, accurate, splice-aware aligner. https://escholarship.org/uc/item/1h3515gn

Ćaleta, M., I. Buj, M. Mrakovčić, P. Mustafić, D. Zanella, Z. Marčić, A. Duplić, T. Mihinjač and I. Katavić (2015). Endemic Fishes of Croatia. Croatian Environment Agency. Zagreb: 1–115.

Ćaleta, M., Z. Marčić, I. Buj, D. Zanella, P. Mustafić, A. Duplić and S. Horvatić (2019). A review of extant Croatian freshwater fish and lampreys – annotated list and distribution. Croatian Journal of Fisheries. 77, 136-232.

Crivelli, A.J. 2006a. *Phoxinellus alepidotus*. *The IUCN Red List of Threatened Species* 2006: e.T39273A10181170. <https://dx.doi.org/10.2305/IUCN.UK.2006.RLTS.T39273A10181170.en>. Accessed on 27 July 2024. Crivelli, A.J. (2006b). *Phoxinellus dalmaticus*. The IUCN Red List of Threatened Species 2006: e.T60761A12395116.

https://dx.doi.org/10.2305/IUCN.UK.2006.RLTS.T60761A12395116.en. Accessed on 13 July 2024.

Crivelli, A.J. 2006c. *Phoxinellus pseudalepidotus*. *The IUCN Red List of Threatened Species* 2006: e.T60826A12415250. <https://dx.doi.org/10.2305/IUCN.UK.2006.RLTS.T60826A12415250.en>. Accessed on 27 July 2024.

Ćurčić V. (1916). Narodno ribarstvo u Bosni i Herecegovini. Glasn. III. Zapadno-bosanski krš, i to ispostava Kupres i kotarevi: Duvno, Glamoć, Livno i Blidinjsko jezero. Glasnik Zemaljskog Muzeja Bosne I Hercegovine, 28, 397–426.

Delić, A., Kučinić, M., Marić, D. i Bučar, M. (2005). New data about the distribution of *Phoxinellus alepidotus* (Heckel, 1843) and *Aulopyge huegelii* (Heckel, 1841). Natura Croatica, 14 (4), 351-355. Preuzeto s https://hrcak.srce.hr/1727

Filipović, M., Frangen, T., Terzić, J, Reberski, L. (2023) Hydrogeology of a complex karst catchment in Southern Dalmatia (Croatia) and Western Herzegovina (Bosnia and Herzegovina). Journal of Maps, 19(1), 2112775. https://doi.org/10.1080/17445647.2022.2112775

Freyhof, J., Lieckfeldt, D., Bogutskaya, N. G., Pitra, C., & Ludwig, A. (2006). Phylogenetic position of the Dalmatian genus *Phoxinellus* and description of the newly proposed genus *Delminichthys* (Teleostei: Cyprinidae). Molecular Phylogenetics and Evolution, 38(2), 416–425. <https://doi.org/10.1016/j.ympev.2005.07.024>

Geiger, M. F., F. Herder, M. T. Monaghan, V. Almada, R. Barbieri, M. Bariche, P. Berrebi, J. Bohlen, M. Casal-Lopez, G. B. Delmastro, G. P. J. Denys, A. Dettai, I. Doadrio, E. Kalogianni, H. Kärst, M. Kottelat, M. Kovačić, M. Laporte, M. Lorenzoni, et al. (2014). Spatial heterogeneity in the Mediterranean Biodiversity Hotspot affects barcoding accuracy of its freshwater fishes. Molecular Ecology Resources 14 (6), 1210–1221.

Jelić, D., Freyhof, J. (2024a) *Phoxinellus alepidotus*. The IUCN Red List of Threatened Species (submitted).

Jelić, D., Freyhof, J. (2024b) *Phoxinellus dalmaticus*. The IUCN Red List of Threatened Species (submitted).

Jelić, D., Freyhof, J. (2024c) *Phoxinellus pseudalepidotus*. The IUCN Red List of Threatened Species (submitted).

Karaman, M. S.  (1972) Süßwasserfische der Türkei. 9. Teil. Revision einiger kleinwüchsiger Cyprinidengattungen *Phoxinellus*, *Leucaspius*, *Acanthobrama* usw. aus Südeuropa, Kleinasien, Vorder-Asien und Nordafrika. Mitteilungen aus dem Hamburgischen Zoologischen Museum und Institut, 69, 115–155.

Marić D. 1980. Prilog poznavanju rasprostranjenja *Paraphoxinus alepidotus* (Heckel, 1843) u vodama Jugoslavije (The contribution to the knowledge of *Paraphoxinus alepidotus* (Heckel, 1843) in waters of Yugoslavia). Glasnik Republičkog Zavoda Zaštite prirode Prirodnjačkog Muzeja, Titograd, 13, 101–105.

Marić D. 1983. Morfološke karakteristike *Paraphoxinus alepidotus* (Heckel, 1843) (Cyprinidae) u ponornice Korane kod Bodanskog Grahova (Jugoslavija). Glasnik Republičkog Zavoda Zaštite prirode Prirodnjačkog Muzeja, Titograd, 16, 65–73.

Markotić, I., Mihaljević, Z., Ćaleta, M. & Glamuzina, B. (2019). Feeding Ecology of the Endemic *Phoxinellus pseudalepidotus* (Cyprinidae) from Mostarsko Blato (Neretva River Basin, Bosnia and Herzegovina). European International Journal of Science and Technology, 8(8), 5–14.

Perea, S.; Böhme, M.; Zupančič, P.; Freyhof, J.; Šanda, R.; Özuluğ, M.; Abdoli, A.; Doadrio, I. Phylogenetic Relationships and Biogeographical Patterns in Circum‐Mediterranean Subfamily Leuciscinae (Teleostei, Cyprinidae) Inferred from Both Mitochondrial and Nuclear Data. BMC Evol. Biol. 2010, 10, 265. https://doi.org/10.1186/1471‐2148‐10‐265.

Roglić, J. (1954). Polja zapadne Bosne i Hercegovine, Prilog poznavanju prirodnih osobina i ekonomskog značenja, Zbornik s Trećeg kongresa geografa Jugoslavije, Sarajevo.

Sabioncello, I. (1967). Sistematika slatkovodnih riba. In: Z. Livojević Z. & C. Bojći (eds.). Pri ručnik za slatkovodno ribarstvo. Agronomski Glasnik, posebna izdanja, 21–90.

Schönhuth, S.; Vukić, J.; Šanda, R.; Yang, L.; Mayden, R.L. (2028) Phylogenetic Relationships and Classification of the Holarctic Family Leuciscidae (Cypriniformes: Cyprinoidei). Molecular Phylogenetics and Evolution, 127, 781–799. https://doi.org/10.1016/j.ympev.2018.06.026.

Štambuk-Giljanović, N. (2002). Vode Cetine i njezina poriječja. Split: Zavod za javno zdravstvo Županije splitsko-dalmatinske, Zagreb: Hrvatske vode. 814 pp.

Taler, Z. (1953). Znamenite i osobite ribe u Bosni i Hercegovini. Ribarski List, (4) XXVIII, Sarajevo, 56–57.

Vuković T. (1982). Sistematika riba. In: C. Bojći et al. (eds.) Slatkovodno Ribarstvo, Poslovna zajednica slatkovodnog ribarstva Jugoslavije. Jugoslavenska medicinska naklada, Zagreb, p. 99–168.

Vuković, T. (1977). Ribe Bosne I Herzigovine. Svjetlost: Sarajevo, Bosnia and Herzegovina.

Vuković, T., Ivanović, B. (1971). Slatkovodne Ribe Jugoslavije. Zemaljski musej BiH-Prirodnjačko odjeljenje: Sarajevo, Bosnia and Herzegovina.

Zupančič, P. (2008). Rare and endangered freshwater fishes of Croatia, Slovenia and Bosnia Hercegovina - Adriatic Basin. Agencija AZV Dolsko d.o.o.: Dolsko, Slovenia. 79 pp.

Zupančič, P. and N. G. Bogutskaya (2000) Description of a new species, *Phoxinellus dalmaticus* (Cyprinidae: Leuciscinae), from the Cikola River in the Krka River system, Adriatic basin (Croatia). Natura Croatica v. 9 (no. 2): 67-81.

Zupančič, P. and N. G. Bogutskaya (2002). Description of two new species, *Phoxinellus krbavensis* and *P. jadovensis*, re-description of *P. fontinalis* Karaman, 1972, and discussion of the distribution of *Phoxinellus* species (Teleostei: Cyprinidae) in Croatia and in Bosnia and Herzegovina. Natura Croatica v. 11 (no. 4): 411-437.
